# The efficacy and safety of apatinib plus capecitabine in platinum-refractory metastatic and/or recurrent nasopharyngeal carcinoma: a prospective, phase II trial

**DOI:** 10.1186/s12916-023-02790-1

**Published:** 2023-03-16

**Authors:** Lin-Quan Tang, Xiao-Yun Li, Zhi-Ming Li, Zhi-Gang Liu, Miao-Zhen Lin, Huan Zhou, Qi-Wen Yu, Jian Zhou, Chong Zhao, Ze-Bin Chen, Xi-Cheng Wang, Jia-Yu Peng, Qiu-Yan Chen, Wen-Feng Fang, Yun-Peng Yang, Bei Zhang, Liang-Ping Xia, Pi-Li Hu, Wei-Han Hu, Yi-Jie Li, Hai-Qiang Mai, Xiu-Yu Cai

**Affiliations:** 1 Department of Nasopharyngeal Carcinoma, Sun Yat-Sen University Cancer Center, State Key Laboratory of Oncology in South China, Guangdong Key Laboratory of Nasopharyngeal Carcinoma Diagnosis and Therapy, Guangzhou, 510060 Guangdong People’s Republic of China; 2 Department of Medical Oncology, Sun Yat-Sen University Cancer Center, Guangzhou, 510060 Guangdong People’s Republic of China; 3 The Cancer Center of the Fifth Affiliated Hospital of Sun Yat-Sen University, Zhuhai, 519000 Guangdong People’s Republic of China; 4 Department of VIP Inpatient, Sun Yat-Sen University Cancer Center, State Key Laboratory of Oncology in South China, Guangdong Key Laboratory of Nasopharyngeal Carcinoma Diagnosis and Therapy, Guangzhou, 510060 Guangdong People’s Republic of China; 5 Department of Oncology, The First Affiliated Hospital of Guangdong Pharmaceutical University, Guangzhou, 510060 Guangdong People’s Republic of China; 6 Department of Medical Imaging, Sun Yat-Sen University Cancer Center, Guangzhou, 510060 Guangdong People’s Republic of China; 7 Department of Radiation Oncology, Sun Yat-Sen University Cancer Center, State Key Laboratory of Oncology in South China, Collaborative Innovation Center for Cancer Medicine, Guangzhou, 510060 Guangdong People’s Republic of China; 8 Jiangsu Hengrui Pharmaceuticals Co., Ltd., Shanghai, People’s Republic of China

**Keywords:** Nasopharyngeal carcinoma, Tyrosine kinase inhibitor, Apatinib

## Abstract

**Background:**

Previous studies have shown that monotherapy with apatinib, an oral tyrosine kinase inhibitor, has promising efficacy for treating recurrent or metastatic (RM) nasopharyngeal carcinoma (NPC) patients. In this study, we aimed to assess the efficacy and safety of apatinib combined with capecitabine as a second-line therapy or beyond for treating RM-NPC patients who failed the first-line platinum-based chemotherapy.

**Methods:**

In this single-arm, phase II study, we enrolled RM-NPC patients who had at least one measurable lesion according to the Response Evaluation Criteria in Solid Tumors (RECIST v1.1). The sample size was determined using Simon’s two-stage design. All patients were administered with apatinib 500 mg once daily and capecitabine 1000 mg/m^2^ twice per day on days 1–14 of each 21-day cycle. The primary endpoint was the objective response rate (ORR), and the secondary endpoints comprised disease control rate (DCR), duration of response (DoR), progression-free survival (PFS), overall survival (OS), and safety.

**Results:**

We enrolled 64 patients from September 2018 to August 2020. The ORR and DCR were 39.1% (95% CI, 27.1–52.1) and 85.9% (95% CI, 75.0–93.4), respectively. The median DoR was 14.4 months (95% CI, 7.8–21.0). As of April 20, 2021, the median follow-up duration was 12.0 months. The median PFS was 7.5 months (95% CI, 5.0–10.0) and the median OS was 15.7 months (95% CI, 11.3–20.1). The most common toxicities of any grade were anemia (75.0%), hand-foot syndrome (65.6%), and proteinuria (64.0%). Grade 3–4 toxicities were observed in 36 (56.3%) patients, with hypertension (14.1%), mucositis (12.4%), and fatigue (10.9%) most commonly observed.

**Conclusions:**

Apatinib plus capecitabine shows promising efficacy as a second-line treatment option in pretreated platinum-refractory RM-NPC patients. Dose selection of this combination needs further investigation considering the toxicity.

**Trial registration:**

Chi-CTR1800017229.

**Supplementary Information:**

The online version contains supplementary material available at 10.1186/s12916-023-02790-1.

## Background

Nasopharyngeal carcinoma (NPC) is a unique subtype of head and neck cancer and endemic in Southern China and Southeast Asia. Radiotherapy with or without chemotherapy is the fundamental treatment strategy for newly diagnosed non-metastatic patients. For metastatic or recurrent patients, platinum-based chemotherapy has been the standard treatment [1]. Among various chemotherapy regimens, the superiority of gemcitabine and cisplatin (GP) over cisplatin and 5-fluorouracil (5-FU) was verified in a phase III trial [2], with an increase in progression-free survival (PFS) from 5.6 to 7.0 months, establishing that regimen as the first-line treatment for recurrent and/or metastatic (RM) NPC. Recently, two multi-center phase III studies verified the superiority of programmed cell death-1 (PD-1) inhibitor camrelizumab [3] and toripalimab [4] over the GP regimen in treating RM-NPC as the first-line treatment, respectively. As reported by Xu et al., a significant improvement in median PFS from 8.0 to 11.7 months was achieved with the addition of toripalimab [4]. However, when the first-line treatment fails, few treatment options remain and a well-established second-line regimen is absent. Therefore, the prognosis of RM-NPC patients is generally poor. In addition, due to the deterioration of nutritional status after cycles of intensive chemotherapy, it is difficult for patients to tolerate another round of conventional chemotherapy. In this setting, more effective salvage options and the establishment of second-line treatment are in urgent need.

In general, single-drug therapy is empirically given in the second-line or subsequent setting, such as 5-FU, paclitaxel, and methotrexate, with reported response rates of 30–40% [5]. Capecitabine is a new-generation oral fluoropyrimidine drug that can be converted into 5-FU to exert cytotoxic anti-tumor activity in cancer tissues and minimize the exposure of 5-FU to normal tissues. The efficacy of capecitabine has been reported by several studies [6–9]. In a recently published phase III study [9], the addition of metronomic adjuvant capecitabine to chemoradiotherapy significantly improved 3-year failure-free survival in patients with high-risk locoregionally advanced NPC. In a phase II study of capecitabine monotherapy in platinum-failed RM head and neck cancer [8], an objective response rate (ORR) of 24.2% and median overall survival (OS) of 7.3 months were achieved, indicating that capecitabine is a feasible alternative for RM patients.

Other combined treatment options include inhibitors of epidermal growth factor receptor (EGFR) and vascular endothelial growth factor (VEGF) and immune checkpoint blockade therapy. To date, in the field of EGFR inhibitors for treating platinum-refractory NPC, little progress has been made, as the results from previous studies failed to achieve satisfactory efficacy due to the small sample size [10, 11]. For the application of immune checkpoint inhibitors, such as PD-1 inhibitors, their anti-tumor activity has been demonstrated, with the reported ORRs of 20.5–34% in pretreated RM-NPC patients [12–15], but it should be noted that the efficacy was also influenced by the expression of programmed cell death-ligand 1 (PD-L1). Anti-angiogenesis therapy is another promising field, and in a phase II trial that evaluated the efficacy of axitinib monotherapy in the second-line or more setting of RM-NPC, the clinical benefit rate reached 78.4% at 3 months and 43.2% at 6 months [16]. In another trial treating metastatic NPC patients with Endostar and cisplatin-based chemotherapy [17], an ORR of 77.8% was achieved, and the median PFS was 12 months, suggesting that the combination of a VEGF inhibitor and chemotherapy might yield synergistic efficacy for the treatment of metastatic NPC.

Apatinib is an oral small molecular tyrosine kinase inhibitor (TKI) that selectively binds with VEGFR2 with high affinity. Its encouraging anti-tumor effect has been shown in studies on various solid tumors, including head and neck cancer [18–23]. In a phase II study treating RM adenoid cystic carcinoma of the head and neck [23], the observed ORR was 46.2% among 65 patients, with a 12-month PFS of 75.2%. Such encouraging results prompted us to explore the efficacy of apatinib in RM-NPC patients and the potential synergistic effect of apatinib plus capecitabine. Therefore, we conducted this phase II trial to assess the efficacy and safety of apatinib in combination with capecitabine for treating first-line-failed platinum-refractory RM-NPC.

## Methods

### Study design and participants

This was a single-arm, open-label phase II study conducted at Sun Yat-sen University Cancer Center (SYSUCC). Eligible patients were aged 18–70 with histologically proven recurrent and/or metastatic NPC and had at least one measurable lesion at baseline according to the Response Evaluation Criteria in Solid Tumors (RECIST) v1.1. All patients should have received at least first-line platinum-containing chemotherapy and progressed after it. Other inclusion criteria included an Eastern Cooperative Oncology Group (ECOG) performance status of 0–2 and adequate hematological, renal, and hepatic functions. Patients were excluded if they had participated in other clinical trials within 4 weeks before the study, had received radiotherapy on the same site for twice or more, or had received the treatment with VEGFR small-molecule TKIs, such as famotidine, sorafenib, sunitinib, regofinib, anlotinib, or fruquintinib. Patients who exhibited a bleeding tendency, including important blood vessel invasion, abnormal coagulation, and/or a history of arterial/venous thrombosis within 6 months, or those with poorly controlled hypertension using a single anti-hypertensive drug were excluded. Patients with multiple factors affecting oral administration and absorption of drugs, such as being unable to swallow, being post-gastrointestinal resection, having chronic diarrhea, and/or intestinal obstruction were also excluded. The detailed inclusion and exclusion criteria can be found in the study protocol.

This study was approved by the Ethics Committee of SYSUCC, and the study was conducted in accordance with the Declaration of Helsinki. All participants provided written informed consent upon enrollment.

### Procedures

At the screening stage, all patients underwent enhanced computed tomography (CT) of the chest, abdomen, and pelvis; enhanced magnetic resonance imaging (MRI) of the head and neck; bone scan; or ^18^F-fluorodeoxyglucose (^18^F-FDG)-PET, and the scanning must be within 28 days before the treatment. The blood, urine, and biochemical routine; coagulation function test; electrocardiogram; Epstein-Barr virus (EBV) DNA level; and blood pressure level should be measured within 7 days before the treatment.

Apatinib was orally administered at 500 mg once per day continuously, and capecitabine was orally administered at 1000 mg/m^2^ twice per day on days 1–14 of each 21-day cycle. The doses of apatinib and capecitabine were chosen based on evidence from previous trials [6, 21, 23]. Treatment was continued until disease progression, intolerant toxicity, death, withdrawal of informed consent, or discontinuity of drugs at the investigator’s discretion. Sequential dose interruption or reduction was permitted for the management of toxicities for any grade 3 or worse adverse events or intolerable grade 2 adverse events evaluated by the clinicians. Drug interruption should not be greater than 2 weeks and the suspension should not exceed two times during each cycle. For both apatinib and capecitabine, two levels of dose reduction were permitted. The reduction levels for apatinib were from 500 mg once daily, to 500 mg and 250 mg on alternate days, to 250 mg once daily. For capecitabine, 25% and 50% dose reductions were given. Once reduced, dose re-escalation was not permitted.

Tumor response was assessed by imaging tests, including enhanced CT or MRI, every two treatment cycles until disease progression or withdrawal for any reason. The response was confirmed by at least one sequential tumor assessment using RECIST v1.1. Blood, urine, and biochemical tests were required at the end of each cycle. All adverse events (AEs) were monitored throughout the treatment and 30 days thereafter and graded according to the National Cancer Institute Common Terminology Criteria for Adverse Events (NCI CTCAE) v4.0.

### Outcomes

The primary endpoint was ORR, defined as the proportion of patients with complete response (CR) and partial response (PR). The secondary endpoints comprised the disease control rate (DCR, defined as the proportion of patients with CR, PR, and stable disease [SD]), duration of response (DoR), PFS, OS, and safety. DoR was defined as the time from the first CR or PR to progressive disease (PD). PFS was defined as the duration from the date of enrollment to the date of any recorded tumor progression or death from any cause, while OS was the duration from the date of enrollment to death from any cause. Patients with neither disease progression nor death were censored at the date of the last follow-up.

### Statistical analysis

This study was a superiority trial using Simon’s two-stage design to estimate the sample size. The one-sided *α* was set as 0.05, and *β* was 0.2, with a testing power of 80%. According to the literature [8], the ORR of capecitabine monotherapy was 24.2%. Assuming that the ORR of the combination of apatinib and capecitabine would increase by 15%, at least 58 cases would be needed to achieve an ORR of 39.2%. During the first stage, 31 cases would be enrolled. If the number of effective cases was fewer than 8, it was considered that the efficacy of the drug combination was not better than that of monotherapy, and the trial would be terminated. Otherwise, the remaining 27 patients would be enrolled in the second stage of enrollment. If 19 of the 58 patients achieved CR or PR, the trial was considered successful; otherwise, the trial protocol was ineffective. Considering the dropout rate of 10%, a total of 64 patients needed to be enrolled.

Efficacy and survival were analyzed in the intention-to-treat (ITT) cohort, which included patients who received at least one cycle of test drugs, and the per-protocol cohort (PP), which excluded those who accepted other anti-tumor treatment during the study period on the basis of the ITT cohort. The safety cohort covered all patients who received at least one cycle of test drugs and had safety records available after medication. The Kaplan–Meier method was used to calculate the DoR, PFS, and OS. The corresponding 95% CIs of ORR and DCR were calculated based on the Clopper-Pearson exact method. In post hoc subgroup analyses, the chi-square test was used to compare ORR in different subgroups. All statistical tests were two-sided, and a *P* value < 0.05 was considered significant. We used SPSS v26.0 for all the analyses. This study was registered on ChiCTR.org.cn, ChiCTR1800017229.

## Results

**Fig. 1:**
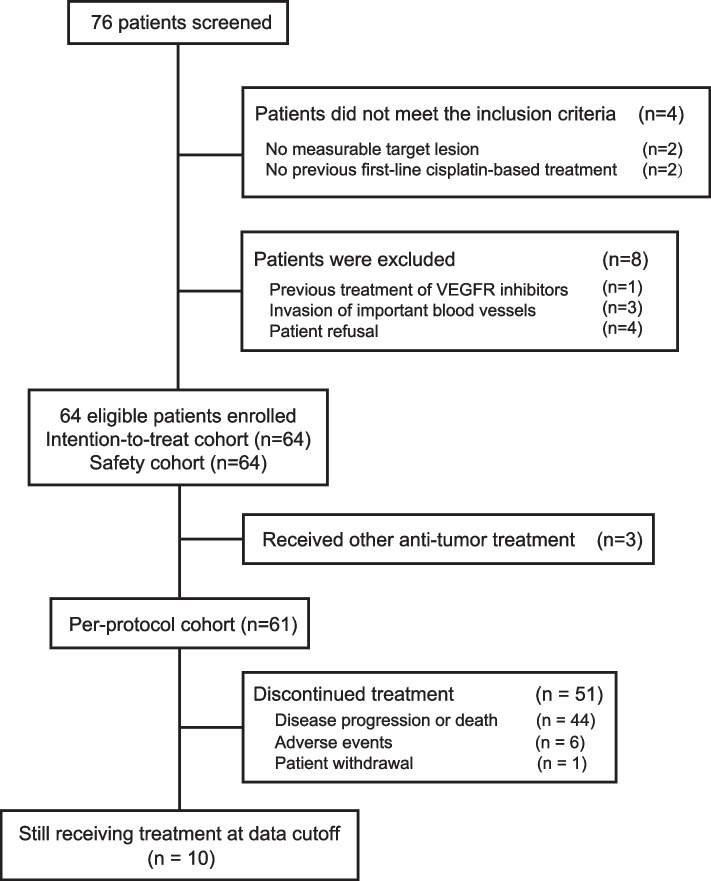
Flow diagram of the trial

From September 2018 to August 2020, a total of 76 patients were screened, 64 of whom met the eligibility requirements and constituted the ITT and safety cohorts (Fig. 1). Additionally, two patients received radiofrequency ablation of pulmonary lesions, and one patient underwent palliative bone radiotherapy; therefore, they were excluded from the PP cohort (*n* = 61). The baseline characteristics are listed in Table 1. Briefly, 59 (92.2%) of 64 patients presented with distant metastases, and 31 (48.4%) patients had metastases with multiple organs involved. Thirty-eight (59.4%) patients had failed first-line cisplatin-based treatment, while 26 (40.6%) patients had failed at least two lines of treatment. Twenty-three (35.9%) patients received PD-1/PD-L1 inhibitor treatment previously. Up to April 20, 2021, the median follow-up duration was 12.0 months (IQR, 7.5–18.3). Ten (15.6%) patients were still on treatment. Nine (14.1%) patients withdrew from the study, among whom 7 were due to AEs and 2 were the patients’ personal decisions.

**Table 1:** Baseline characteristics

Characteristic, *n* (%)	Intention-to-treat population (*n* = 64)
Gender	
Male	49 (76.6)
Female	15 (23.4)
Age, median (IQR), years	44 (39–54)
Histology	
WHO I/II	8 (12.5)
WHO III	56 (87.5)
ECOG performance status	
0	29 (45.3)
1	19 (29.7)
2	16 (25.0)
Recurrence and/ or metastasis	
Recurrence	5 (7.8)
Metastasis	44 (68.8)
Recurrence and metastasis	15 (23.4)
Involved metastatic organs	
Single	28 (43.8)
Multiple	31 (48.4)
Location of metastases	
Liver	30 (46.9)
Lung	26 (40.6)
Bone	22 (34.4)
Others	24 (37.5)
Target lesion size, median (IQR), mm	47 (33–79)
Plasma EBV DNA level, median (IQR), copies/mL	3915 (422–32,500)
No. of previous treatment	
1	38 (59.4)
2	12 (18.8)
≥ 3	14 (21.9)
Previous PD-1/PD-L1 inhibitor treatment	
Yes	23 (35.9)
No	41 (64.1)

*Abbreviations*: *ECOG*, Eastern cooperative oncology group, *EBV* Epstein-Barr virus, *PD-1*, programmed cell death-1, *PD-L1* Programmed cell death-ligand 1

### Efficacy

**Fig. 2:**
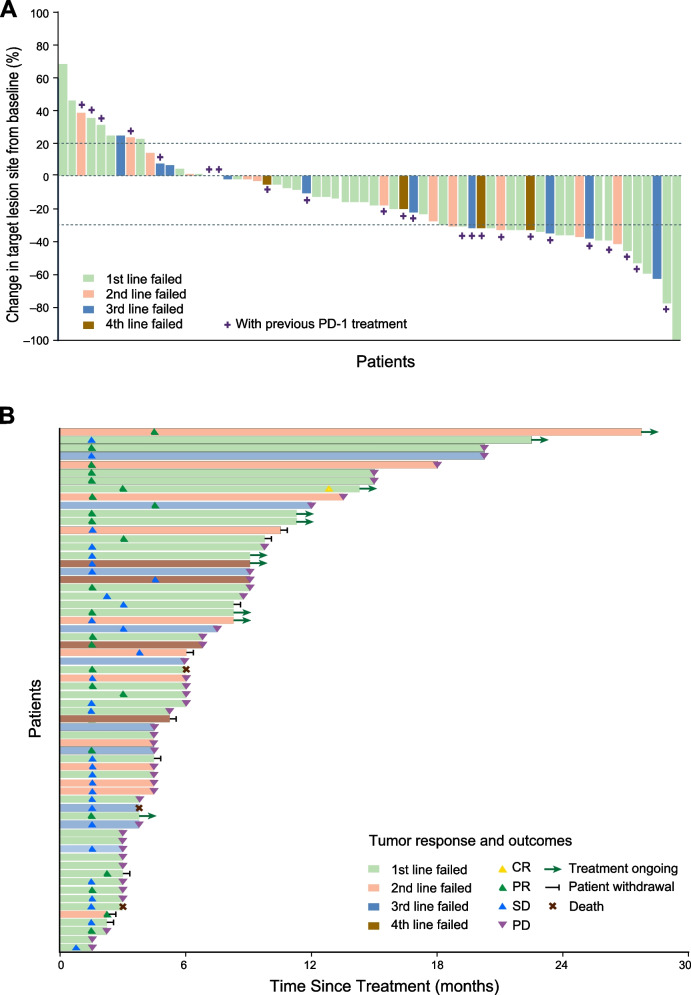
Tumor response assessment according to the RECIST v1.1. **A** Best percentage change from baseline in target lesion. **B** Duration of responses

Among the 31 patients enrolled in the first stage, 12 patients achieved PR, which was above the pre-defined threshold for the first stage of Simon’s two-stage. In the ITT cohort, PR was observed in 25 patients, of whom one patient achieved CR after receiving 17 cycles of treatment. The ORR was 39.1% (95% CI, 27.1–52.1), and the median time from enrollment to response was 1.5 months (2 cycles). Meanwhile, 30 (46.9%) patients had SD, with a DCR of 85.9% (95% CI, 75.0–93.4). The ORR and DCR in the PP cohort were 41.0% (95% CI, 28.6–54.3) and 85.2% (95% CI, 73.8–93.0), respectively (Table 2; Fig. 2). Among patients with confirmed CR or PR, the median DoR was 14.4 months (95% CI, 7.8–21.0), and the response was maintained for over 12 months in 15 (40.0%) patients. Representative images of therapeutic efficacy are shown in Additional file 1: Figs. S1-2.

**Table 2:** Efficacy and survival in the intention-to-treat and per-protocol cohorts

	**Intention-to-treat population (*n* = 64)**		**Per-protocol population (*n* = 61)**	
	**No. (%) or %**	**95% CI**	**No. (%) or %**	**95% CI**
**Response**				
CR	1 (1.6)	-	1 (1.6)	-
PR	24 (37.5)	-	24 (39.3)	-
SD	30 (46.9)	-	27 (44.3)	-
PD	9 (14.1)	-	9 (14.8)	-
**ORR**	39.1	27.1–52.1	41.0	28.6–54.3
**DCR**	85.9	75.0–93.4	85.2	73.8–93.0
**Progression-free survival**				
Median, months	7.5	5.0–10.0	7.5	5.1–9.9
6-month rate	57.8	45.6–70.0	57.4	45.1–69.7
12-month rate	37.5	25.3–49.7	36.1	23.8–48.4
**Overall survival**				
Median, months	15.7	11.3–20.1	13.4	9.0–17.8
6-month rate	81.3	71.6–90.9	82.0	72.4–91.6
12-month rate	65.6	53.6–77.6	65.6	53.3–77.9

Response was assessed according to the RECIST v1.1. Only confirmed responses were listed

*Abbreviations*: *CR* Complete response, *PR* Partial response, *SD* Stable disease, *PD* Progressive disease, *ORR* Objective response rate, *DCR* Disease control rate

**Fig. 3:**
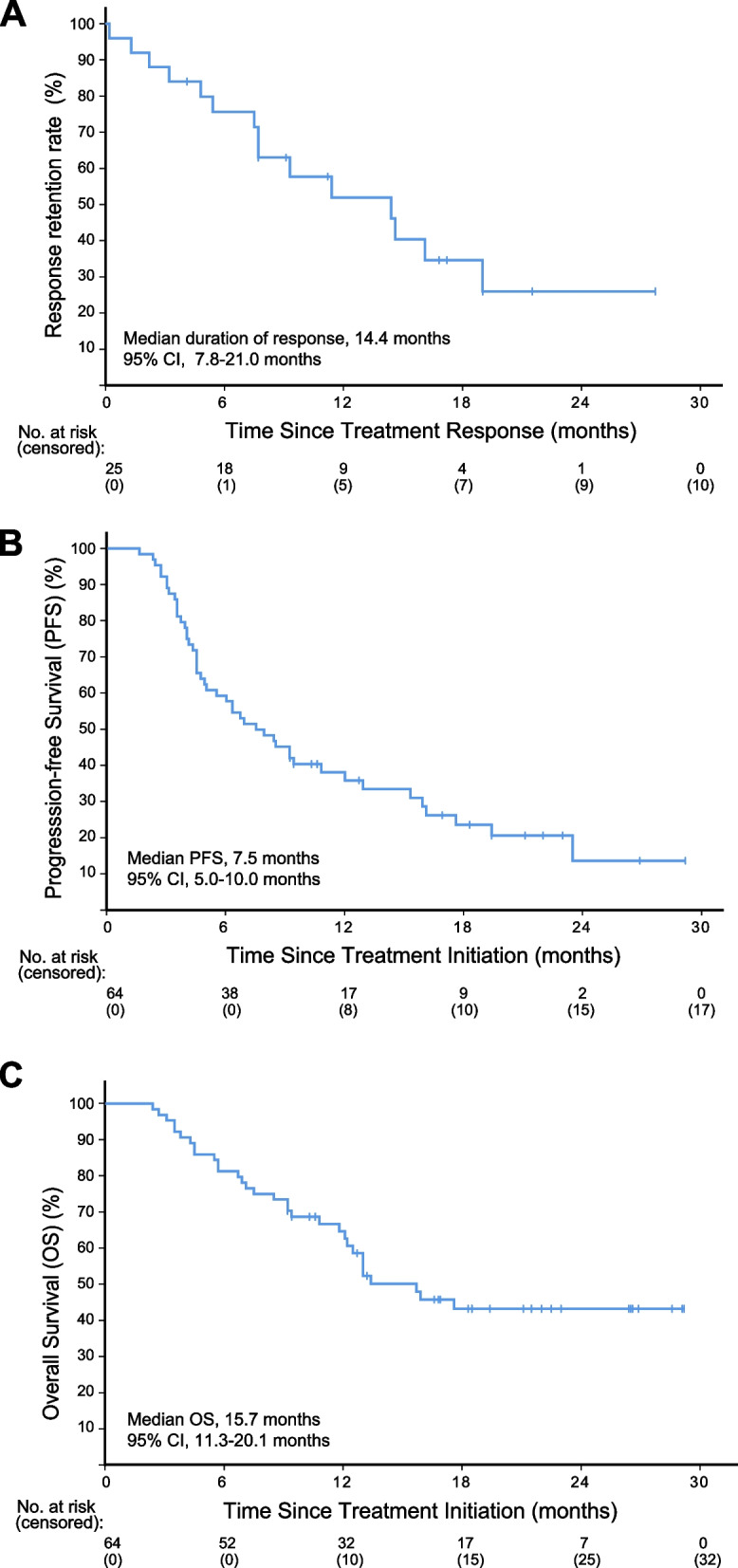
Kaplan–Meier curves showing the duration of response (**A**), progression-free survival (**B**), and overall survival (**C**) in the ITT cohort (*n* = 64)

Forty-seven (73.4%) patients suffered from disease progression, with a median PFS of 7.5 months (95% CI, 5.0–10.0). The 6-month PFS was 57.8% (95% CI, 45.6–70.0%). Twenty-nine (45.3%) patients died of cancer, while 3 (4.7%) patients died of treatment-related AEs (two nasal hemorrhage and one pulmonary infection). The median OS was 15.7 months (95% CI, 11.3–20.1). The 6-month and 12-month OS rates were 81.3% (95% CI, 71.6–90.9%) and 65.6% (95% CI, 53.6–77.6%), respectively (Table 2; Fig. 3).

### Safety

All patients in the safety cohort reported at least one occurrence of AEs. The most common toxicities of any grade were anemia (75.0%), hand-foot syndrome (65.6%), and proteinuria (64.0%). Thirty-six patients (56.3%) suffered from grade 3–4 toxicities, with grade 3 hypertension (14.1%), mucositis (12.4%), and fatigue (10.9%) most commonly observed. A grade 4 AST increase was recorded in one patient (Table 3). Six (9.3%) patients withdrew from the study due to treatment-related toxicities, including grade 3 fatigue (two patients), grade 3 proteinuria, grade 3 dysphagia, pulmonary atelectasis, and acute upper gastrointestinal hemorrhage.

**Table 3:** Cumulative acute adverse events in safety population (*n* = 64)

	**Grade 1–2**	**Grade 3**	**Grade 4**
**Hematologic**			
Anemia	43 (67.2)	5 (7.8)	0
Leukopenia	33 (51.6)	0	0
Neutropenia	24 (37.5)	0	0
Thrombocytopenia	11 (17.2)	4 (6.2)	0
**Non-hematologic**			
Hand-foot syndrome	40 (62.5)	2 (3.1)	0
AST increased	38 (59.4)	0	1 (1.6)
Proteinuria	37 (57.8)	4 (6.2)	0
Fatigue	33 (51.6)	7 (10.9)	0
Total bilirubin increased	29 (45.3)	4 (6.2)	0
Mucositis	28 (43.8)	8 (12.4)	0
Vomiting	25 (39.1)	1 (1.6)	0
Nausea	24 (37.5)	0	0
Hyponatremia	22 (34.4)	6 (9.3)	0
Hemorrhage	21 (32.8)	2 (3.1)	0
Hypocalcemia	21 (32.8)	0	0
Hypertension	20 (31.3)	9 (14.1)	0
Hypoalbuminemia	19 (29.7)	0	0
Diarrhea	17 (26.6)	3 (4.7)	0
Creatinine increase	16 (25.0)	0	0
Rash	11 (17.2)	0	0
Allergic reactions	11 (17.2)	0	0
Anorexia	10 (15.6)	0	0
Constipation	10 (15.6)	0	0
ALT increased	10 (15.6)	1 (1.6)	0
Hypokalemia	8 (12.4)	2 (3.1)	0
Fever	7 (10.9)	1 (1.6)	0
Hoarseness	5 (7.8)	0	0

Data are *n* (%)

*ALT* Alanine aminotransferase, *AST* Aspartate aminotransferase

The median cycles of apatinib and capecitabine administered were 8 cycles (IQR, 5–9). Dose reduction of apatinib occurred in 47 (73.4%) patients, of whom 15 patients (31.9%) required two levels of reduction, and 14 patients (21.9%) received a full dose of the combination. The most frequent causes of apatinib dose reduction were mucositis (25/62, 40.3%) and hemorrhage (13/62, 21.0%). In regard to capecitabine, 33 (51.5%) patients experienced dose reduction, with the hand-foot syndrome (11/39, 28.2%) and poor overall performance status (8/39, 20.5%) being the most common causes (Additional file 2: Fig S3-5). The median period of the first dose reduction of apatinib occurred in the second cycle, with a median duration of 46 days, compared with the third cycle and 52 days for capecitabine, respectively.

Three patients died of serious AEs; two patients died of epistaxis and one died of pulmonary infections. The two patients who died of grade 3 nasal hemorrhage had tumors invasion into cavernous sinuses at the time of diagnosis and received at least 5 cycles of treatment. Therefore, the death was considered to be partially related to the study drug. For the patient who died of pulmonary infection, we lost contact with her after 4 cycles of treatment and learned that she died 2 months after the last dose, the death was considered to be irrelated to the study drug. The details of the deaths can be found in Additional file 3 (Table S1).

### Post hoc analysis

We performed a post hoc analysis to see whether the efficacy of apatinib plus capecitabine was affected by patient characteristics and previous treatment. Patients with multiple organ involvement exhibited a much lower ORR than those with metastasis in a single organ (54.5% vs. 22.6%, *P* = 0.009). However, EBV DNA level before treatment was not associated with tumor response. Meanwhile, no difference in ORR was observed between the second-line and multi-line subgroups. ORR did not differ between subgroups with or without previous PD-1/PD-L1 inhibitor treatment (Additional file 4, Fig. S6).

## Discussions

To our knowledge, this is the first study assessing the efficacy and safety of apatinib plus capecitabine for treating RM-NPC in the second-line or more settings. Our primary endpoint was met with a confirmed objective response observed in 39.1% of patients. Most toxicities were manageable with clinical interventions, dose reduction, and interruption, suggesting that this combination therapy is a feasible option for platinum-refractory RM-NPC.

Angiogenesis is the hallmark of tumorigenesis and is promoted by the expression of VEGF. Reported overexpression of VEGF in the NPC microenvironment is the foundation of the application of anti-angiogenesis drugs in NPC [24]. Before apatinib, various VEGF inhibitors were studied in NPC in different clinical settings, including sorafenib, pazopanib, famitinib, axitinib, etc. However, only a modest response was observed in these studies. For instance, in the studies of pazopanib and famitinib [10, 25], ORRs ranged from 6.1 to 8.6%. For axitinib, the unconfirmed ORR was 18.9%, while the confirmed ORR was 2.7% [16]. The addition of anti-angiogenic therapy to chemotherapy in RM-NPC has also been explored. In a study that treated chemotherapy-naïve RM-NPC with sorafenib plus cisplatin and 5-FU, the ORR reached 77.8% with a median PFS of 7.2 months [26]. Nonetheless, it did not significantly improve efficacy compared to chemotherapy alone. Apatinib is more advantageous than other TKIs due to its enhanced selectivity and binding affinity [27, 28]. Apatinib monotherapy was administered to treat platinum-refractory RM-NPC, and the reported ORRs were 31.4–36.4%% [29–31]. Moreover, apatinib in combination with chemotherapy also exhibits promising therapeutic effects, as demonstrated in a study that treated platinum-refractory metastatic NPC with apatinib and S-1, with an ORR of 34.1% and a DCR of 80.4% reached [32]. In our study, the ORR and DCR were 39.1% and 85.9%, respectively, indicating that the combination of apatinib and capecitabine might convey superior anti-tumor effects to other regimens. It was also noted that in post hoc analysis, patients with metastasis in a single organ exhibited a significantly higher ORR than those with multiple organ involvement (54.5% vs. 22.6%, *P* = 0.009), suggesting the applicability of this regimen in patients with a low tumor burden. In addition, apatinib and capecitabine are both cost-effective orally administered drugs. This combination therapy, which requires no hospitalization, is particularly applicable in patients with poor overall status or those who want to maintain a normal life.

In recent years, immunotherapy has made great progress in treating different solid tumors, including NPC. Compared to the trials that assessed the anti-tumor activity of different PD-1 antibody monotherapies in pretreated RM-NPC, the ORR from our study was slightly higher (39.1% vs. 20.5–34%), as was the median PFS (7.5 months vs. 1.9–6.5 months) [12–15]. In terms of median OS, the results were comparable across studies (15.7 months from our study vs. 16.5–17.4 months) [13–15]. In addition, when performing post hoc analysis to compare the efficacy of apatinib and capecitabine in patients with or without a previous history of PD-1/PD-L1 antibodies, the ORR in PD-1/PD-L1 inhibitor-pretreated patients was higher than that in patients without (47.8% vs. 34.1%). Though the *P* value was not significant, which might be due to the small sample size, we believe it is a field that is worth further exploration, along with the impact of PD-L1 expression on the efficacy of apatinib. Given that the vessel normalization induced by anti-angiogenic agents ameliorates tumor hypoxia and facilitates the immune functions of immunocytes, studies on other cancers have explored the feasibility of implementing a dual blockade of VEGF and immune checkpoints [33, 34]. Encouragingly, promising outcomes were observed, and our trial assessing the efficacy of camrelizumab plus apatinib is currently ongoing (NCT04548271).

The recommended dose for apatinib in gastric cancer was 850 mg [35], while in the studies of apatinib monotherapy for NPC, the initial dose of apatinib was usually set as 500 mg [29–31]. Considering that the impaired drug absorption rate might lead to a heavier dose of the drug in gastric cancer patients after surgery, and the combination of apatinib and capecitabine might exaggerate the treatment toxicity, we chose 500 mg as the initial dose in the present study. The dose reduction of apatinib was observed in 73.4% of patients, which was higher than previously reported rates of 42.9–60.8% in studies on apatinib monotherapy [29, 30], but is consistent with the results observed in cervical cancer [36]. Given the high incidence of dose reduction in this study, a lower initial dose of apatinib should be considered. Several studies have provided reference for the appropriate dose of apatinib. In a study of apatinib combined with oral etoposide in platinum-resistant/refractory ovarian cancer, dose reductions of apatinib occurred in 28 (82%) of 34 patients, of whom 16 patients had dose reductions from 500 to 250 mg, but this combination still showed promising PFS and DoR [37]. Another study by Wang et al. showed that the initial dose of 250 mg apatinib combined with pegylated liposomal doxorubicin (PLD) reached a longer median PFS than the PLD alone group (5.8 months vs. 3.3 months) in platinum-resistant recurrent ovarian cancer [38]. Therefore, 250 mg may be a suitable initial dose of apatinib in combination therapy. Regarding acute toxicities, most of which in this study showed higher incidences than those in studies on apatinib monotherapy [29, 30], such as mucositis and hand-foot syndrome, which might be explained by the addition of capecitabine, and the overall poor performance status of patients in our study. In addition, the dose of capecitabine may also be considered for adjustment, given the surprising efficacy of metronomic capecitabine in nasopharyngeal and breast cancers published recently [39, 40]. In the phase III clinical trial of Chen et al., the addition of low-dose metronomic administration of 650 mg/m^2^ capecitabine as an adjuvant therapy demonstrated a significantly failure-free survival benefit in patients with high-risk locoregionally advanced nasopharyngeal carcinoma [39]. Besides, metronomic capecitabine also exhibited lower toxicity and better tolerability. Therefore, a combination of metronomic capecitabine and 250 mg apatinib may be a regimen worth exploring. Among grade 3 toxicities, hypertension, mucositis, and fatigue were most frequently observed in this study, in accordance with previous studies [19, 21]. Similar to other TKI drugs [41, 42], nasal hemorrhage has always been a critical issue when treating NPC patients. The incidence of hemorrhage was 35.9% in our study, but the majority of them were grade 1, with no treatment cessation required. Two patients experienced grade 3 hemorrhage and died from nasal hemorrhage. Both deaths had tumor invasion into cavernous sinuses at the time of diagnosis, and it warned us to be more prudent when deciding candidates for apatinib. Patients with vascular involvement (confirmed or suspicious) and local recurrence should be excluded.

The current study has several limitations. This was a single-arm study with no comparative group; therefore, the comparison of efficacy between this study and historical data lacked power. The enrolled patients were heterogenous, including both the recurrent and metastatic patients, and some of them had received multiple lines of chemotherapy. Besides, as confined by the small sample size, we were unable to find out which patients benefited more from the combination therapy. We did not collect quality of life prospectively due to the lack of expectation of AEs. Meanwhile, certain difficulties also existed in the collection of patient-reported outcomes in the outpatient clinic. Therefore, we could not know whether the high toxicity of apatinib plus capecitabine at the study dose level affects patients’ quality of life. However, given the high proportion of dose reduction and high incidence of toxicities, lower initial doses of apatinib and capecitabine should be considered in future studies.

## Conclusions

In conclusion, the current findings suggest that apatinib plus capecitabine shows promising efficacy in pretreated platinum-refractory RM-NPC patients. This combination deserves further investigation in this population, but the dose selection needs reconsideration.

## Supplementary Information

**Additional file 1: Figures S1-S2.** Fig.S1-Serial computed tomography scans showing the appearance of the target lesions before treatment. Fig.S2-Serial computed tomography scans showing the appearance of the target lesions after 6 weeks (two cycles) of treatment.

**Additional file 2: Figures S3-S5.** Fig S3- Dose reduction of experimental drugs. Fig S4- Main causes for reduction of apatinib. Fig S5- Main causes for reduction of capecitabine.

**Additional file 3: Table S1.** Information on death cases

**Additional file 4:** **Figure S6.** Fig.S6- Post hoc analysis of potential factors influencing the efficacy of apatinib plus capecitabine.

**Additional file 5. **Clinical Trial Protocol

## Data Availability

Supplementary material related to this article can be found in the online version.

## Abbreviations

^18^F-FDG-PET: ^18^F-fluorodeoxyglucose positron emission tomography
5-FU: 5-Fluorouracil
AEs: Adverse events
CR: Complete response
CT: Computed tomography
DCR: Disease control rate
DoR: Duration of response
EBV-DNA: Epstein-Barr virus DNA
ECOG: Eastern Cooperative Oncology Group
EGFR: Epidermal growth factor receptor
GP: Gemcitabine and cisplatin
ITT: Intention-to-treat
MRI: Magnetic resonance imaging
NCI CTCAE: National Cancer Institute Common Terminology Criteria for Adverse Events
NPC: Nasopharyngeal carcinoma
ORR: Objective response rate
OS: Overall survival
PD-1: Programmed cell death protein 1
PFS: Progression-free survival
PLD: Pegylated liposomal doxorubicin
PP: Per-protocol
PR: Partial response
RECIST: Response evaluation criteria in solid tumors
RM-NPC: Recurrent or metastatic nasopharyngeal carcinoma
SD: Stable disease
SYSUCC: Sun Yat-sen University Cancer Center
TKI: Tyrosine kinase inhibitor
VEGF: Vascular endothelial growth factor
