# Supplementary figures and images for: The efficacy and safety of apatinib plus capecitabine in platinum-refractory metastatic and/or recurrent nasopharyngeal carcinoma: a prospective, phase II trial
Source: BMC Med. 2023 Mar 16;21:94. doi: 10.1186/s12916-023-02790-1 (PMC10022300; doi:10.1186/s12916-023-02790-1)

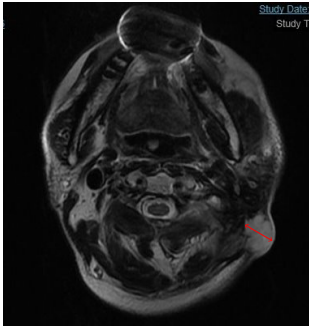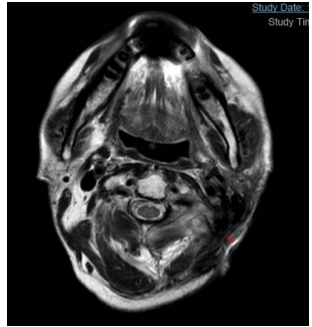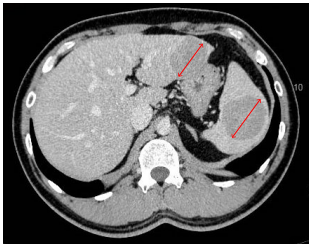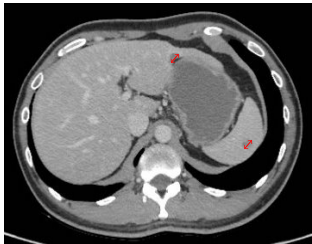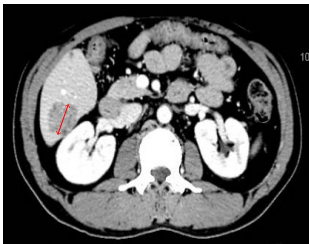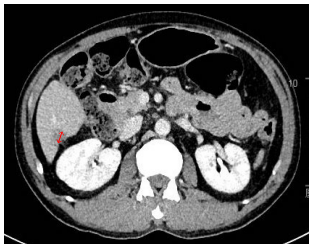

**S1.**Before treatment

**S2.**Two cycles after treatment

Supplement: Supplementary file 1 — Additional file 1: Figures S1-S2. Fig.S1-Serial computed tomography scans showing the appearance of the target lesions before treatment. Fig.S2-Serial computed tomography scans showing the appearance of the target lesions after 6 weeks (two cycles) of treatment. [file 12916_2023_2790_MOESM1_ESM.pdf]

**S3**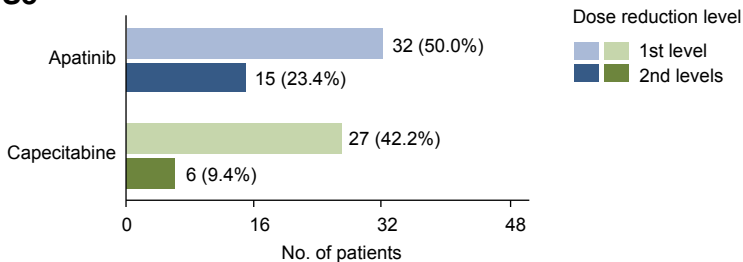**S4**

Causes for dose reduction of Apatinib

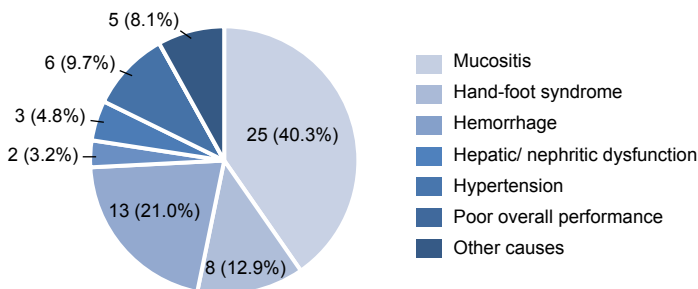**S5**

Causes for dose reduction of Capecitabine

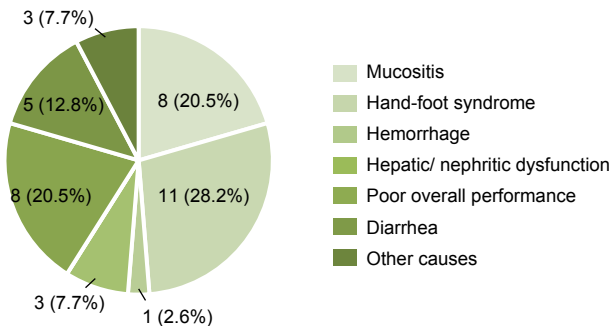

Supplement: Supplementary file 2 — Additional file 2: Figures S3-S5. Fig S3- Dose reduction of experimental drugs. Fig S4- Main causes for reduction of apatinib. Fig S5- Main causes for reduction of capecitabine. [file 12916_2023_2790_MOESM2_ESM.pdf]

**S6**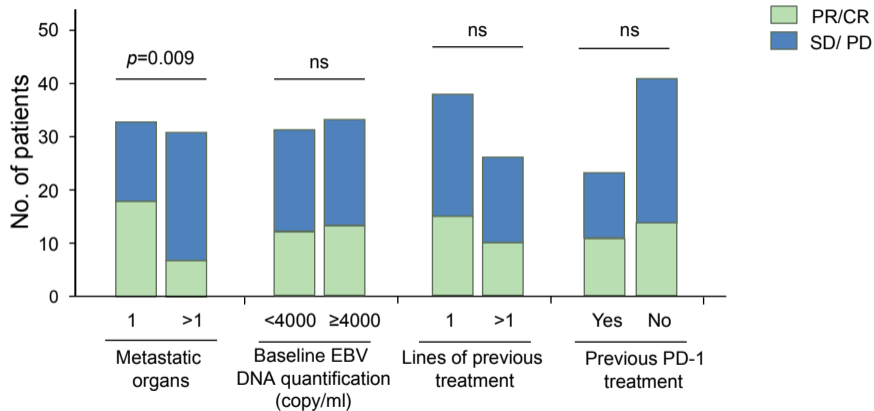

Supplement: Supplementary file 4 — Additional file 4: Figure S6. Fig.S6- Post hoc analysis of potential factors influencing the efficacy of apatinib plus capecitabine. [file 12916_2023_2790_MOESM4_ESM.pdf]
