# Supplementary material for: The efficacy and safety of apatinib plus capecitabine in platinum-refractory metastatic and/or recurrent nasopharyngeal carcinoma: a prospective, phase II trial
Source: BMC Med. 2023 Mar 16;21:94. doi: 10.1186/s12916-023-02790-1 (PMC10022300; doi:10.1186/s12916-023-02790-1)
Supplement: Supplementary file 3 — Additional file 3: Table S1. Information on death cases [file 12916_2023_2790_MOESM3_ESM.docx]

**Table S1. Information on death cases**

|  | Case 1 | Case 2 | Case 3 |
| --- | --- | --- | --- |
| Age, years | 42 | 60 | 44 |
| Sex | Male | male | Female |
| Date of diagnosis | 2015/7/20 | 2017/12/22 | 2017/8/12 |
| Pathological type | Nonkeratinizing, undifferentiated subtype | Nonkeratinizing, undifferentiated subtype | Nonkeratinizing, undifferentiated subtype |
| Disease characteristics | Nasopharyngeal MR showed that the tumor had invaded bilateral cavernous sinuses. Extensive destruction of basicranial. | Nasopharyngeal MR showed that the tumor had invaded the left cavernous sinuses.  pet-ct showed: multiple lung metastases | No distant metastasis. |
| Prior treatment | 1.Concurrent chemoradiotherapy  2.Docetaxel+5-Fu+ cisplatin | 1.Concurrent chemoradiotherapy,  2.Tegafur+cyclophosphamide  3. Toripalimab | 1.Concurrent chemoradiotherapytreatment  2.Gemcitabine + vinorelbine + nimotuzumab  3.Albumin paclitaxel + carboplatinand  4.A phase I clinical trial of EGFR-targeted drugs MRG003 |
| Enrollment date | 2018/9/12 | 2018/4/19 | 2019/8/10 |
| Overall lesion diameter at baseline | 54 mm | 84mm | 131mm |
| Apatinib and capecitabine Treatment | 2018/9/27-2018/12/26(4 cycles) | 2018/4/27-2019/8/31(6 cycles) | 2019/6/26-2019/8/10 (1 cycle, lost contact after 2019/8/11) |
| Death date | 2018/12/27 | 2019/9/1 | 2019/10 |
| Death reasons | Nasal hemorrhage | Nasal hemorrhage | Pulmonary infection |
| Relevance to the study drug | Partially related | Partially related | Irrelevant |
