# Supplementary material for: The efficacy and safety of apatinib plus capecitabine in platinum-refractory metastatic and/or recurrent nasopharyngeal carcinoma: a prospective, phase II trial
Source: BMC Med. 2023 Mar 16;21:94. doi: 10.1186/s12916-023-02790-1 (PMC10022300; doi:10.1186/s12916-023-02790-1)
Supplement: Supplementary file 5 — Additional file 5. Clinical Trial Protocol [file 12916_2023_2790_MOESM5_ESM.docx]

**A Phase II Study to Evaluate the Efficacy and Safety of Apatinib in Combination with Capecitabine in Patients with Recurrent/Metastatic Nasopharyngeal Carcinoma** **Who Failed Platinum-based Therapies**

**Clinical Trial Protocol**

**Investigator: Xiu-Yu Cai, Hai-Qiang Mai**

**Sun Yat-sen University Cancer Center**

**Sponsor: Jiangsu Hengrui Medicine Co., Ltd.**

**Protocol Version : 2.2**

**Date of Version: 29 October 2018**

**Table of Contents**

[PROTOCOL SIGNATURE 1](#_Toc76113653)

[PROTOCOL SYNOPSIS 2](#_Toc76113654)

[SCHEMA 6](#_Toc76113655)

[1. STUDY BACKGROUND 7](#_Toc76113656)

[2. STUDY OBJECTIVES 10](#_Toc76113662)

[2.1. Primary Objective 11](#_Toc76113663)

[2.2. Secondary Objective 11](#_Toc76113664)

[3. STUDY DESIGN 11](#_Toc76113665)

[3.1. Study Design Description 11](#_Toc76113666)

[3.2. Study Drug 11](#_Toc76113667)

[3.2.1. Medication Regimen 11](#_Toc76113668)

[3.2.2. Distribution and recycling of drugs 11](#_Toc76113669)

[3.3. Sample Size Estimation 12](#_Toc76113670)

[3.4. Criteria for Inclusion, Exclusion and Withdrawal of subjects 12](#_Toc76113671)

[3.4.1. Inclusion Criteria 12](#_Toc76113672)

[3.4.2. Exclusion Criteria 13](#_Toc76113673)

[3.4.3. Removal Criteria 14](#_Toc76113674)

[3.4.4. Withdrawal Criteria 14](#_Toc76113675)

[3.4.5. Protection of Subjects' Rights and Interests 14](#_Toc76113676)

[3.5. Concomitant Treatment 15](#_Toc76113677)

[3.5.1. Hematology Support 15](#_Toc76113678)

[3.5.2. Treatment of Non-hematological Toxicity Symptoms 15](#_Toc76113679)

[3.5.3. Others 15](#_Toc76113680)

[3.6. Dose Delay and Adjustment 15](#_Toc76113681)

[3.7. Symptomatic Treatment of Common Adverse Reactions of Apatinib and Capecitabine 16](#_Toc76113682)

[3.7.1. Hypertension 16](#_Toc76113683)

[3.7.2. Proteinuria 16](#_Toc76113684)

[3.7.3. Hand-Foot Skin Reaction 17](#_Toc76113685)

[3.7.4. Skin Rash 18](#_Toc76113686)

[3.7.5. Bleeding 18](#_Toc76113687)

[3.7.6. Mouth Ulcers 18](#_Toc76113688)

[3.7.7. Bone Marrow Suppression 18](#_Toc76113689)

[3.7.8. Nausea, Vomiting and Loss of Appetite 19](#_Toc76113690)

[3.7.9. Hepatic Function Impairment 19](#_Toc76113691)

[3.8. Study Procedures 19](#_Toc76113692)

[3.8.1. Screening Stage 19](#_Toc76113693)

[3.8.2. Treatment Stage 21](#_Toc76113694)

[3.8.3. End of Treatment/Withdrawal from the Study 22](#_Toc76113695)

[3.8.4. 30-day Follow-up after Subject Withdrawal 23](#_Toc76113696)

[3.8.5. Survival Follow-up 23](#_Toc76113697)

[3.9. Efficacy Evaluation 23](#_Toc76113698)

[3.9.1. Measuring Method 23](#_Toc76113699)

[3.9.2. Record Tumor Measurement Data 23](#_Toc76113700)

[3.9.3. Efficacy Evaluation Indicators 24](#_Toc76113701)

[3.9.3.1. Primary Efficacy Indicators 24](#_Toc76113702)

[3.9.3.2. Secondary Efficacy Indicators 24](#_Toc76113703)

[3.9.4. Safety Evaluation 24](#_Toc76113704)

[3.9.5. Serious Adverse Event (SAE) 25](#_Toc76113705)

[3.10. Ethical, Regulations and Administrative Principles 27](#_Toc76113706)

[3.10.1. Ethical Principles 27](#_Toc76113707)

[3.10.2. Informed Consent 27](#_Toc76113708)

[3.10.3. Data Protection 28](#_Toc76113709)

[3.10.4. Confidential Agreement 28](#_Toc76113710)

[3.10.5. Record Keeping 28](#_Toc76113711)

[3.10.6. Early Suspension of The Study 28](#_Toc76113712)

[3.10.7. Inspection by Sponsor and The Regulatory Authority 28](#_Toc76113713)

[3.11. Data Collection and Management 29](#_Toc76113714)

[3.11.1. Data Entry and Modification 29](#_Toc76113715)

[3.11.2. Data Lock 29](#_Toc76113716)

[3.11.3. Statistical analysis Data Set 29](#_Toc76113717)

[3.11.4. Statistical Analysis Plan 29](#_Toc76113718)

[3.11.5. Quality Control and Quality Assurance 30](#_Toc76113719)

[References 31](#_Toc76113720)

[Appendix 1 AJCC/UICC 7th Edition TNM Staging 33](#_Toc76113721)

[Appendix 2 ECOG PS Scoring 34](#_Toc76113722)

[Appendix 3 Flow Chart of the Study 35](#_Toc76113723)

#

# PROTOCOL SIGNATURE

**Investigator Agreement:**

I confirm I have read this protocol. I agree to conduct this study in arccordance with the Declaration of Helsinki and to comply with and the moral, ethical and scientific principles stipulated by China's Good Clinical Practice (GCP), and to conduct this study according to the design and regulatory requirements of this protocol.

I will be in charge of making medical decisions related to the clinical trial to ensure the subjects can receive timely treatment when an adverse event (AE) occurs during the trial. I am fully aware of the correct procedures and requirements for reporting serious adverse events (SAE) and I will record and report such events as required.

I guarantee that the data will be loaded into the Case Record Form (CRF) in an accurate, complete, timely and legitimate manner. I will accept the supervision and inspection from the supervisor or inspector dispatched by the sponsor, and the drug regulatory authority to ensure the quality of the clinical trial.

I agree to use the study results for drug registration.

I will provide a resume before the commencement of this study and submit to the Ethics Committee and probably the drug administration department.

Investigator (signature): Date:

# PROTOCOL SYNOPSIS

| **Study Drug** | Apatinib plus Capecitabine |
| --- | --- |
| **Study Title** | A Phase II Study to Evaluate the Efficacy and Safety of Apatinib in Combination with Capecitabine in Patients with Recurrent/Metastatic Nasopharyngeal Carcinoma Who Failed Platinum-based Therapies |
| **Investigator** | Xiuyu Cai; Haiqiang Mai |
| **Protocol Version** | 2.2 |
| **Protocol Date** | October 29, 2018 |
| **Study Design** | A non-randomized, open-label, single-arm exploratory study |
| **Study Properties** | A clinical study initiated by the investigator |
| **Study Subjects** | Patients with recurrent/metastatic advanced nasopharyngeal carcinoma (NPC) who failed platinum-based therapies |
| **Study Objective** | To evaluate the efficacy and safety of apatinib combined with capecitabine in the treatment of patients with recurrent/metastatic advanced NPC who failed platinum-based therapies |
| **Study Sample Size** | This study is a superiority trial using Simon's two-stage design to estimate the sample size. Alpha is 0.05, Beta is 0.2, and the testing power is 0.8. According to literature, the ORR of capecitabine monotherapy is 24.2%, assuming that the ORR of the combination of apatinib and capecitabine increases by 15%, i.e., ORR = 39.2%, and the drop-out rate of 10%, at least 64 cases are needed. In the first stage, 31 cases will be enrolled, when the number of effective cases is less than 8, it is considered that the efficacy of the drug combination is not better than the monotherapy, and the trial will be terminated. Otherwise, continue to the second stage of enrollment. |
| **Subject Eligibility Criteria: Inclusion Criteria** | Patients to be included in this study must meet all of the following inclusion criteria:  1) Patients with histologically proven recurrent and/or metastatic NPC;  Patients with recurrent and/or metastatic NPC shall meet any of the following items: 1. Recurrence after radiotherapy; 2. Metastasis  2) Patients who have failed the platinum-based therapy and received at least first-line platinum-containing chemotherapy;  3) Male or female patients: aged 18-70 y/o;  4) ECOG Performance Status score: 0-2;  5) The estimated survival is ≥ 3 months;  6) Patients not receiving chemotherapy or radiotherapy 4 weeks before the inclusion;  7) With at least one measurable lesion according to the RECIST criteria (the size measured with MRI or spiral CT scan is ≥ 10 mm and the measurable lesion has not received any radiotherapy);  8) Patients whose major organs function well, i.e., to meet the following requirements 1 week before the inclusion:  a. Routine blood test:  i. Hemoglobin > 80 g/L (no blood transfusion within 14 days);  ii. Neutrophil count > 1.5 × 10^9^/L;  iii. Blood platelet count > 80 × 10^9^/L;  b. Biochemical examination:  i. Total bilirubin ≤ 1.5 × ULN (upper limit of normal);  ii. ALT or AST ≤ 2.5 × ULN (upper limit of normal);  iii. Creatinine < 2.0 × ULN, endogenous creatinine clearance rate > 50 ml/min (Cockcroft-Gault Equation);  9) Females of child-bearing age must undergo a pregnancy test (serum or urine) 7 days before the inclusion, with a negative result and a willingness to take reliable contraceptive methods during the trial;  10) Subjects voluntarily participate in the study, sign the Informed Consent form, and are well compliant and cooperative in follow-ups. |
| **Subject Eligibility Criteria: Exclusion Criteria** | Patients that meet any of the following items shall not be included in this study:  1) Patients who are allergic to any of the drugs in this study;  2) Pregnant or lactating females;  3) Patients who have participated in clinical trials of other drugs within 4 weeks before this study;  4) Patients who have received radiology for twice or more times;  5) Patients who have received the treatment with VEGFR small-molecule tyrosine kinase inhibitors (such as famotidine, sorafenib, sunitinib, regofinib, anlotinib, fruquintinib);  6) Patients whose MRI images show tumors have invaded important blood vessels (such asthe surrounding internal carotid artery/vein); or patients who are highly likely to experience fatal hemorrhage due to the potential impact of tumors on important vessels, as judged by the investigators;  7) Patients with severe hemorrhage history and undergoing any hemorrhage event of Grade 3 or above in CTCAE (Version 4.0) within 4 weeks before the inclusion;  8) Hypertensive patients who cannot be well controlled by a single anti-hypertensive drug (systolic blood pressure > 140 mmHg, diastolic blood pressure > 90 mmHg); cardiovascular disease with clinical significance (e.g. active), such as cerebrovascular accident (≤ 6 months before randomization), myocardial infarction (≤ 6 months before randomization), unstable angina pectoris, congestive heart failure with the NYHA classification of Class II or above, or severe arrhythmias that cannot be controlled with drugs or have a potential impact on the trial treatment;  9) Patients with active ulcer, intestinal perforation and intestinal obstruction;  10) Patients with a digestive tract perforation history 28 days before the selection;  11) With multiple factors affecting oral administration and absorption of drugs (such as being unable to swallow, after gastrointestinal resection, chronic diarrhea and intestinal obstruction);  12) Patients with abnormal coagulation and bleeding tendency (14 days before signing the Informed Consent form, the following conditions must be met: INR is within a normal range without the use of anticoagulants); patients treated with anticoagulants or Vitamin K antagonists such as warfarin and heparin or similar drugs; under the premise that the INR of the prothrombin time is ≤ 1.5, small doses of warfarin (1 mg, oral administration, once a day) or small doses of aspirin (the daily dosage shall not exceed 100 mg) are permitted for preventive purposes;  13) Patients suffering from arterial/venous thrombosis events within 6 months before the screening, such as cerebrovascular accidents (including transient ischemic attacks), deep vein thrombosis (except for patients who had venous thrombosis caused by intravenous catheterization due to previous chemotherapy and are judged to have recovered by the investigators) and pulmonary embolism;  14) Patients with renal insufficiency: Urinary routine proteinuria > 2+ and the urinary protein quantification of 24 hours is confirmed to be > 1.0 g;  15) Patients undergoing major surgery within 28 days;  16) Patients who have received the treatment with potent CYP3A4 inhibitors 1 week before the inclusion or received the treatment with potent CYP3A4 inductive agents within 2 weeks before participating in this study;  17) Patients with chronic unhealed wounds or incompletely healed fractures;  18) Patients with symptomatic brain metastasis (confirmed or suspected);  19) Patients with severe or uncontrolled infection;  20) Patients with a history of psychotropic substance abuse and unable to quit or with mental disorder history;  21) Patients with a history of immunodeficiency diseases, including HIV-positive, or with other acquired or congenital immunodeficiency diseases, or with organ transplant history;  22) Patients with past or present objective evidence for pulmonary fibrosis, interstitial pneumonia, pneumoconiosis, radioactive pneumonia, drug-related pneumonia, severe pulmonary function impairment;  23) Patients with a history of other malignant tumors in the past 5 years, except for cured skin basal cell carcinoma, cervical carcinoma in situ and superficial bladder cancer;  24) Concomitant diseases that seriously endanger patients' safety or affect patients' completion of this study, as judged by the investigators. |
| **Drug Dosing and Administration Regimen** | Apatinib Mesylate Tablets 500 mg, po, qd, q4w, plus Capecitabine tablets 1000 mg/m^2^, po, bid, d1-14, q3w (take the medicine at the same time every day as possible);  A treatment cycle lasts 3 weeks, and the combined medication will be continued until the occurrence of toxicity intolerance, disease progression, or death, or patients refuse to continue participating in this clinical study, or the investigators determine that the medication must be terminated. |
| **Study Efficacy Evaluation** | **Intent-to-Treat (ITT) Population**: The ITT population is defined as all subjects who are enrolled.  **Per Protocol (PP) Population**: The per protocol population (PP) defines as a subset of the subjects who meet all of the trial criteria and are compliant with the protocol and absence of any major protocol violations.  The efficacy is assessed by imaging examinations and the RECIST 1.1 criteria will be employed:  **Primary efficacy indicators:** Objective response rate (ORR) of tumor  **Secondary efficacy indicators:**   1. Disease control rate (DCR) 2. Duration of response (DoR) 3. Patient's progression-free survival (PFS) 4. Overall survival (OS)   5) Safety and tolerance of the regimen |
| **Study Safety Evaluation** | All the patients that have received the treatment with the test drugs for at least one cycle, and have safety records will be taken as an effective population for safety analysis. The abnormal values of patients' physical examination results, vital signs, adverse events and laboratory examinations will be summarized. Adverse events will be reported and classified according to the Common Terminology Criteria for Adverse Events (CTCAE) Version 4.0 of the NCI. |
| **Study Statistical Methods** | Efficacy evaluation:  The 95%CI of ORR and DCR are calculated based on the Clopper-Pearson exact method.  The Kaplan-Meier method is used to estimate the median DOR, PFS and OS, and survival curves will be created.  Safety evaluation:  Safety evaluation is mainly based on descriptive statistical analyses and lists the adverse events and adverse reactions occurring in this trial (adverse reactions are defined as "adverse events that are 'definitely related/probably related/likely related to' the test drugs"). The laboratory examination results describe circumstances that are normal before the test but abnormal after the treatment, as well as their relationships with the test drugs when an abnormal change occurs. |

# SCHEMA

Disease progression

Capecitabine (1000 mg/m^2^ bid po d1-14 q3w)

Apatinib (500 mg qd po)

(A treatment cycle lasts 21 days)

Sign the Informed Consent

Screening, check and inclusion

Patients with platinum-failure recurrent/metastatic NPC

Every 2 treatment cycles after taking the drugs, imaging examinations will be performed to assess the efficacy

Apatinib treatment will be terminated and survival follow-up will continue

Tumor reduction or stabilization

The original treatment regimen will be continued until the occurrence of disease progression or intolerable adverse reactions

# 1. STUDY BACKGROUND

Nasopharyngeal carcinoma (NPC) is one of the most common head and neck tumors in Southeast Asia, ranking first among malignant head and neck tumors in China, especially in southern China, including Guangdong, Guangxi, Fujian, Jiangxi and Hong Kong, where the NPC incidence is the highest in the world. For patients with early and locally advanced NPC, the standard treatment regimen is synchronous chemo-radiotherapy, with a five-year survival rate of 80% ^[1]^. However, there are still 8.6%-23.7% of patients who have a recurrence of nasopharyngeal and cervical lymph nodes within five years and need retreatment, and 65%-85% of patients relapse within three years after the first course of treatment ^[2]^. Besides, the lesion site of NPC is hidden and hard to notice, so 70% of patients are already at the advanced stage when discovered clinically, and the most majority of them have developed distant metastases, leading to poor prognosis.

## 1.1. There is No Standard Second-line Chemotherapy Regimen for Advanced Recurrent or Metastatic NPC

Due to the low incidence of NPC in Western countries, there is insufficient high-quality research to support the treatment of advanced NPC. So far, the platinum-containing chemotherapy based on cisplatin combined with 5-Fu has been used in early-stage patients, often serving as the first-line chemotherapy regimen. Other active drugs include paclitaxel, docetaxel, capecitabine, irinotecan, vinorelbine, gemcitabine and ifosfamide, which can be used in combination or monotherapy. The need for continuous venous perfusion and the poor convenience of the commonly used drug 5-Fu can easily cause venous infection, thrombosis and oral mucositis, which seriously affect the quality of life of patients.

In a phase II clinical study, Chen et al. ^[3]^ applied the triplet combination (TP) regimen as the first-line treatment for patients with recurrence and metastasis, achieving a response rate of 79%, with the mPFS and mOS being 8.6 months and 22.7 months, respectively. There was also a repor about the combination of lobaplatin with docetaxel as first-line chemotherapy for recurrent and metastatic NPC, which achieved an overall remission rate (ORR) of 61.5% and a mPFS of 10.0 months ^[4]^.

On August 23, 2016, *The Lancet* published online the results of a phase 3 clinical study by Prof. Zhang Li and his team on gemcitabine plus cisplatin versus fluorouracil plus cisplatin for recurrent or metastatic NPC. The objective response rate of the GP group vs. the FP group was 64% vs 42%, the mPFS was 7.0 months vs. 5.6 months, the mOS was 29.1 months vs. 20.1 months, the risk of disease progression dropped by 45%, the risk of death decreased by 38%, and the total reverse reaction rate in the two groups was similar ^[5]^. Prof. Zhang Li's study provides new clinical data support for the first-line medication regimen for patients with recurrent or metastatic NPC.

The prognosis of recurring or metastatic NPC patients after the failure of first-line platinum-based therapy is poor. No well-established second-line or third-line regimens are available. Meanwhile, many patients are generally in bad conditions after the failure of platinum-based therapy and unable to tolerate combination chemotherapy due to the deterioration of ECOG score and nutritional status. In most cases, such patients become platinum-resistant, so it is difficult for them to continue to benefit from platinum-containing combination chemotherapy regimens. Clinically, single-drug therapy is often preferred, such as fluorouracil, paclitaxel and methotrexate.

Capecitabine is a new-generation oral fluoropyrimidine drug that can be rapidly absorbed gastrointestinally in the original form. It can be converted into 5-Fu with antitumor activity in the liver and cancer tissues to exert a cytotoxic effect. Thymidine phosphorylase (TP), the last enzyme in the activation process of capecitabine, can make 5-FU selectively activated in the tumor tissue and minimize the systemic exposure of 5-FU, thereby avoiding the damage of 5-FU to normal tissues. Peng et al. ^[6]^ treated recurrent or metastatic NPC patients with the combination chemotherapy of capecitabine and nedaplatin after the failure of the cisplatin-based first-line chemotherapy，the response rate reached 41.7%, mPFS and mOS were 5.8 months and 12.4 months, respectively. In another clinical study using capecitabine monotherapy to treat 49 advanced NPC patients (71% with distant metastasis), CR was 6%, PR was 31%, mPFS was 5 months, and mOS was 14 months ^[7]^. According to a study published in *British Journal of Cancer* in 2010, capecitabine monotherapy for patients with recurrent or metastatic head and neck squamous cell carcinoma^[8]^ was effective in 33 out of 40 cases, including 2 cases of CR, 6 cases of PR, 18 cases of SD, and mTTP was 4.8 months, mOS was 7.3 months, and ORR was 24.2%, which indicated that capecitabine could serve as an effective and safe drug to treat patients with recurrent or metastatic head and neck squamous cell carcinoma.

There are many options for palliative chemotherapy for recurrent or metastatic NPC, but most relevant studies have a small sample size, and there are neither sufficient large-scale prospective research data nor sufficient separate research data on recurrent NPC and distant metastasis. The research and development of high-efficiency, highly selective, low-toxicity and convenient therapies are urgently needed.

## 1.2. EGFR Inhibitors Have Proven Efficacies, but the High Price Limits Their Clinical Application

EGFR is a transmembrane glycoprotein that belongs to the HER family. The binding of its extracellular part to EGF can activate the tyrosine kinase in the cell membrane, thereby regulating cell growth and differentiation. The high expressions of EGFR and relevant ligand transforming growth factors are closely associated with the high invasiveness of tumors, high lymph node metastasis, short-term recurrence and survival. The expression of EGFR in different tumors varies. The positive rate of EGFR expression in NPC patients is significantly higher than that in the control group, and the EGFR positive rate increases as the differentiation of tumors becomes worse. The EGFR expression in NPC patients with cervical lymph node metastasis is notably higher than that in those without cervical lymph node metastasis ^[9, 10]^, indicating there is a certain relationship between EGFR and the occurrence of NPC, cancer cell differentiation and tumor progression.

Nimotuzumab is a monoclonal antibody drug targeting EGFR, and the effective rate of nimotuzumab in combination with radiotherapy in the treatment of advanced NPC can reach up to more than 90%. The Chinese version of the NCCN Guidelines for Head and Neck Cancers has recommended nimotuzumab as one of the regimens combining radiotherapy for NPC in 2010 ^[11]^. Cetuximab is an FDA-approved first-line therapeutic drug for recurrent/metastatic head and neck squamous cell carcinoma. Chan et al. ^[12]^ treated 60 recurrent/metastatic advanced NPC patients resistant to platinum treatment using cetuximab combined with carboplatin, and the results showed 20% of CR, 80% of PR, 100% of DCR, and 7.7 months of mOS 7.7. A retrospective study in 2016 ^[13]^ showed that among 30 recurrent or metastatic NPC patients treated with cetuximab in combination with concurrent chemo-radiotherapy (chemotherapy regimens including TP/TPF and GP/PC, etc.), 21 cases had clinical responses (70%), mOS was 23.6 months, PFS was 12.2 months, and the two-year OS reached 53.3%. Though cetuximab demonstrates good efficacy, the expensive treatment price, especially in developing countries, limits its clinical application.

## 1.3. PD-1 Antibody Immunotherapy Shows Preliminary Efficacy and Needs further Exploration

Chen et al. ^[14]^ reported that PD-L1 was expressed in 89% of EBV-associated NPC. A study on EVB-associated NPC ^[15]^ also found that PD-L1 was mainly expressed in EBV-positive NPC cells and a better local control rate could be achieved in patients with low PD-L1 expression. A retrospective study analyzed the relationship between the expression of PD-1/PD-L1 in NPC patients and the post-treatment outcome ^[16]^. Among the 139 advanced NPC patients receiving chemotherapy, it was found through tissue immunohistochemistry that 37.4% (52/139) of the cases had PD-1 expression and 95% (132/139) of the cases had PD-L1 expression, the DFS of patients with high PD-L1 expression was worse than that of patients with low PD-L1 expression (p = 0.009), and the DFS of patients with co-expression of PD-1 and PD-L1 was even worse (p = 0.038). The results suggested that PD-1/PD-L1 expressions could predict recurrence and metastasis of NPC after treatment, and blocking this pathway provided a potential therapy target for NPC.

On August 24, 2018, *JCO* published online a phase Ib clinical study on the use of Pembrolizumab in treating recurrent or metastatic NPC patients with PD-L1 expression ≥ 1% (keynote-028). 27 patients received pembrolizumab treatmentand were followed up for 20 months (median). The results showed that there were 7 cases of PR, 14 cases of SD, 25.9% of ORR, 8 cases (29.6%) of SAEs (Grade 3 and above) occurred including 1 case of drug-related death (sepsis) ^[17]^. Meanwhile, there are ongoing phase II clinical studies on the use of Nivolumab in advanced recurrent/metastatic NPC and the combined use of Nivolumab and Ipilimumab in EBV-positive advanced NPC.

## 1.4. Antiangiogenesis is An Important Target for Treating NPC

Angiogenesis, primarily regulated by vascular growth factors and vascular growth inhibitory factors, plays a vital role in the growth and metastasis of tumors. There are more than 20 vascular growth factors including EGF, VEGF and PDGF, among which VEGF is the most important one. VEGF binds to its receptor, activates the VEGFR-mediated downstream signal pathway, promotes mitosis and chemotaxis of endothelial cells, increases vascular permeability, and causes the extravasation of fibrinogen in blood vessels, which not only provides the essential matrix for endothelial cell migration and the establishment of neonatal capillary network, but also provides raw materials for tumor growth. Studies have shown that VEGF is over-expressed in 67% of NPC patients, and the high expression of VEGF is significantly correlated with the high recurrence rate of NPC and the survival of patients ^[18]^. The efficacy of antiangiogenic therapy in NPC has been verified to a certain extent.

A multicenter phase II clinical study ^[19]^ found that adding bevacizumab to standard chemo-radiotherapy for locally advanced NPC patients can delay the progression of subclinical NPC, and this regimen did not cause hemorrhage in Grade 3-4 patients. During the follow-up of 2.5 years (median), the two-year PFS rate was 74.7%, the two-year distant metastasis-free survival (DMFS) rate was 90.8%, and the two-year OS was 90.9%. Therefore, bevacizumab demonstrates certain advantages in both PFS and OS. According to a study where the recurrent or metastatic head and neck cancer/NPC patients were treated with sorafenib monotherapy ^[20]^, among 27 cases, there were 1 case of PR and 10 cases of SD, and mOS was 4.2 months. This indicated the limited efficacy of the sorafenib monotherapy and a combination with chemo-radiotherapy might be required. In another study of sorafenib combined with cisplatin and fluorouracil ^[21]^, 54 recurrent or metastatic NPC patients were treated with the combination therapy, mPFS was 7.2 months and mOS was 11.8 months, suggesting that the combination of sorafenib with chemotherapy is a feasible regimen for the treatment of metastatic NPC. A single-arm exploratory study of single-agent sunitinib [22] found that 10 out of 14 patients received imaging assessment at least once and tumor reduction was found in 5 cases, but 2 cases with tumor invasion of the carotid sheath died of hemorrhage during the experiment, and the possible cause might be the rupture of large cervical vessels during tumor regression.

Li Bin et al. ^[23]^ treated 30 metastatic NPC patients with endostar combined with chemotherapy (cisplatin + fluorouracil or gemcitabine + cisplatin) and found 4 cases of CR, 22 cases of PR, 3 cases of SD, 1 case of PD, clinical benefit rate of 96.7%, and objective response rate of 86.7%, suggesting that endostar combined with chemotherapy had good short-term efficacy and tolerability in the treatment of metastatic NPC. At CSCO 2015 International Antiangiogenic Therapy Forum, Professor Han Fei and his team from Sun Yat-Sun University Cancer Center reported that among 22 patients who used Endostar combined with radiotherapy/chemotherapy in treating locally advanced recurrent NPC, 20 patients had CR and 2 patients had PR, with the tumor response rate up to 100% and the two-year OS of 66.4%, and that the combined therapy reduced the occurrence of nasopharyngeal mucosa necrosis, but did not shorten the OS, and to some extent improved the quality of life of recurrent NPC patients after treatment.

## 1.5. Apatinib may be a Potentially Effective Drug for Advanced NPC

Apatinib Mesylate is invested and developed by Jiangsu Hengrui Medicine Co., Ltd., with independent intellectual property rights. It is a small molecular tyrosine kinase inhibitor that mainly blocks the signaling pathway of VEGF binding to its receptors by highly selective inhibition of VEGFR-2 (vascular endothelial cell growth factor receptor 2) activity, thereby strongly inhibiting tumor angiogenesis and playing an anti-tumor effect. As a novel oral small molecular tyrosine kinase inhibitor, Apatinib can effectively inhibit VEGFR at very low concentrations, with the IC50 value of 2nM at the VEGFR2 target, 45 times that of Sorafenib and 5 times that of Sunitinib, while at high concentrations, it can also inhibit the platelet-derived growth factor receptor (PDGFR) and c-Kit and c-Src kinases.

The study of the subgroup analysis of Apatinib in a phase IV study on gastric cancer (Ahead-G201) published by Qin Shukui et al. ^[24]^ on ASCO-GI in 2018 showed that patients given 500 mg qd of the drug had fewer both total AEs and grade 3 to 4 AEs than those given 850 mg qd (P = 0.001), the mPFS of the two groups was 4.63 and 2.23 months, respectively (P = 0.0459), and the mOS was 6.83 and 4.04 months, respectively (P = 0.0083), proving that Apatinib at the dose of 500mg qd was sufficiently safe and effective in the real world.

Peng Qiuxia et al. ^[25]^ also found in NPC cell line CNE-2 transplantation tumor model that the combined use of Apatinib and cis-platinum could significantly inhibit tumor growth, extend survival, reduce VEGFR-2 expression, decrease microvessel density, with better efficacy than Apatinib alone. Moreover, VEGFR-2 was an important predictive marker for the efficacy of Apatinib in treating NPC. According to the study by Lin Yansong et al. ^[26]^, 10 patients with iodine-refractory differentiated thyroid carcinoma had their OOR and DCR up to 90% and 100% after receiving 750 mg qd of Apatinib monotherapy (*oncotarget*, 201702), and the main adverse reactions included hypertension, hand-foot-skin reactions and hypocalcemia. In 2017 *EMBO*, He Xiaohui et al. ^[27]^ reported a phase II prospective study on Apatinib monotherapy in the treatment of head and neck metastatic adenocarcinoma after multiple chemotherapies, and found that the ORR of 8 patients was 25%, the DCR was 87.5%, and the drug toxicity tolerable and controllable. Zhu Guopei et al. ^[28]^ from Shanghai Ninth People's Hospital reported the results of a phase II clinical study on Apatinib treating recurrent or metastatic adenoid cystic carcinoma at the 2018 Multidisciplinary Head and Neck Cancers Symposium (AHNS/ASTRO/ASCO), a total of 38 patients received 500 mg of Apatinib alone had an mPFS of 4.5 months, an ORR of 44.7%, and a DCR of 89.5%, suggesting the huge potential efficacy of Apatinib in treating head and neck tumors.

# 2. STUDY OBJECTIVES

The study objectiveis to evaluate the efficacy and safety of Apatinib plus Capecitabine combination therapy in treating patients with recurrent/metastatic NPCwho failed platinum-based therapies.

## 2.1. Primary Objective

The primary objective is to evaluate the objective response rate (ORR) of Apatinib plus Capecitabine combination therapy in treating patients with recurrent/metastatic NPC who failed platinum-based therapies.

## 2.2. Secondary Objective

Secondary objectives are disease control rate (DCR), duration of response (DOR), the progression-free survival (PFS), overall survival (OS), as well as the safety and tolerance of the therapy.

# 3. STUDY DESIGN

## 3.1. Study Design Description

This is a non-randomized, single-arm, open phase II clinical study to evaluate the efficacy and safety of Apatinib combined with Capecitabine therapy in treating patients with recurrent/metastatic nasopharyngeal carcinoma who failed platinum-based therapies.

If a patient does not withdraw the Informed Consent form, is not intolerant to the toxic side effects of the drug, or is not believed unsuitable for further trials by the investigator, he/she should continue the study and treatment until tumor progression with solid imaging evidence. Efficacy indicator and safety indicator will be observed in the study.

## 3.2. Study Drug

### 3.2.1. Medication Regimen

Oral medication of Apatinib of 500 mg, qd, with 4 weeks as a cycle; oral medication of Capecitabine of 1,000 mg/m^2^ once after breakfast and once after dinner every day for two consecutive weeks, with three weeks as a cycle.

With 3 weeks as a treatment cycle, patients will be given both drugs until PD, intolerant toxicity, death, withdrawal of the Informed Consent form, or discontinuity of drugs at the investigator's judgment.

### 3.2.2. Distribution and recycling of drugs

The study drug shall be uniformly stored, distributed and recycled by the study unit according to the GCP requirements. The drugs should be distributed and kept by special personnel, with strict management, and the receipt and use of them should be recorded in detail by the investigator during each visit.

When distributing drug to a patient on the first day of each cycle, from the first day of the first cycle when the patient receives free medication, the investigators should:

- State the number of Apatinib tablets to be distributed to the patient in the next treatment cycle;

- Fill such information (the number of Apatinib tablets to be distributed) in the CRF page of the study drug, and record in the drug distribution logbook.

Instruct patient to obey the treatment regimen, and return all unused tablets and all drug packages (including empty boxes and remaining tablets) at each visit to the hospital.

The total quantity of the trial drugs will be 120% of the designed dose. The investigator must ensure that all study drugs are used only in subjects participating in the clinical trial, and unused drugs will be returned to the sponsor, Jiangsu Hengrui Medicine Co., Ltd. after the end of the trial, with remaining drugs and empty drug packages to be collected by the monitors. The study drugs cannot be given to anyone not participating in the clinical trial.

## 3.3. Sample Size Estimation

This study is a superiority trial using Simon's two-stage design to estimate the sample size. One-sided alpha is 0.05, beta is 0.2, and the testing power is 0.8. According to literature, the ORR of capecitabine monotherapy is 24.2%, assuming that the ORR of the combination of apatinib and capecitabine increases by 15%, i.e., ORR = 39.2%, given a 10% drop-out rate, at least 64 cases are needed. In the first stage, 31 cases will be enrolled, when the number of effective cases is ≤ 7, it is considered that the efficacy of the drug combination is not better than the monotherapy, and the trial will be terminated. Otherwise, continue to the second stage of enrollment.

## 3.4. Criteria for Inclusion, Exclusion and Withdrawal of subjects

### 3.4.1. Inclusion Criteria

1) Patients with histologically proven recurrent and/or metastatic NPC;

Patients with recurrent and/or metastatic NPC shall meet any of the following: 1. Recurrence after radiotherapy; 2. Metastasis

2) Patients who have failed the platinum-based therapy and received at least first-line platinum-containing chemotherapy;

3) Male or female patients: aged 18-70 y/o;

4) ECOG Performance Status score: 0-2;

5) The estimated survival is ≥ 3 months;

6) Patients not receiving chemotherapy or radiotherapy 4 weeks before the inclusion;

7) With at least one measurable lesion according to the RECIST criteria (the size measured with B-mode ultrasound, MRI or spiral CT scan is ≥ 10 mm and the measurable lesion has not received any radiotherapy);

8) Patients whose major organs function well, i.e., to meet the following requirements 1 week before the inclusion:

a. Blood routine examination:

i. Hemoglobin > 80 g/L (no blood transfusion within 14 days);

ii. Neutrophil count > 1.5 × 10^9^/L;

iii. Blood platelet count > 80 × 10^9^/L;

b. Biochemical examination:

i. Total bilirubin ≤ 1.5 × ULN (upper limit of normal);

ii. ALT or AST ≤ 2.5 × ULN (upper limit of normal);

iii. Creatinine < 2.0 times the ULN, endogenous creatinine clearance rate > 50 ml/min (Cockcroft-Gault Equation);

9) Females of child-bearing age must undergo a pregnancy test (serum or urine) 7 days before the inclusion, with a negative result and a willingness to take reliable contraceptive methods during the trial;

10) Subjects voluntarily participate in the study, sign the Informed Consent form, and are well compliant and cooperative in follow-ups.

### 3.4.2. Exclusion Criteria

1) Pregnant or lactating female;

2) Patients who have participated in clinical trials of other drugs within 4 weeks before this study;

3) Patients who have received radiotherapy for twice or more times;

4) Patients who have received the treatment with VEGFR small-molecule tyrosine kinase inhibitors (such as famotidine, sorafenib, sunitinib, regofinib, anlotinib, furosemide);

5) Patients whose MRI images show tumors have invaded important blood vessels (such as the surrounding internal carotid artery/vein); or patients who are highly likely to experience fatal hemorrhage due to the potential impact of tumors on important vessels, as judged by the investigators;

6) Patients with severe hemorrhage history and undergoing any hemorrhage event of Grade 3 or above in CTCAE (Version 4.0) within 4 weeks before the inclusion;

7) Hypertensive patients who cannot be well controlled by a single anti-hypertensive drug (systolic blood pressure > 140 mmHg, diastolic blood pressure > 90 mmHg); cardiovascular disease with clinical significance (e.g. active), such as cerebrovascular accident (≤ 6 months before randomization), myocardial infarction (≤ 6 months before randomization), unstable angina pectoris, congestive heart failure with the NYHA classification of Class II or above, or severe arrhythmias that cannot be controlled with drugs or have a potential impact on the trial treatment;

8) Patients with active ulcer, intestinal perforation and intestinal obstruction;

9) Patients with a digestive tract perforation history 28 days before the selection;

10) With multiple factors affecting oral administration and absorption of drugs (such as being unable to swallow, after gastrointestinal resection, chronic diarrhea and intestinal obstruction);

11) Patients with abnormal coagulation and bleeding tendency (14 days before signing the Informed Consent form, the following conditions must be met: INR is within a normal range without the use of anticoagulants); patients treated with anticoagulants or Vitamin K antagonists such as warfarin and heparin or similar drugs; under the premise that the INR of the prothrombin time is ≤ 1.5, small doses of warfarin (1 mg, oral administration, once a day) or small doses of aspirin (the daily dosage shall not exceed 100 mg) are permitted for preventive purposes;

12) Patients suffering from arterial/venous thrombosis events within 6 months before the screening, such as cerebrovascular accidents (including transient ischemic attacks), deep vein thrombosis (except for patients who had venous thrombosis caused by intravenous catheterization due to previous chemotherapy and are judged to have recovered by the investigators) and pulmonary embolism;

13) Patients with renal insufficiency: Urinary routine proteinuria > 2+ and the urinary protein quantification of 24 hours is confirmed to be > 1.0 g;

14) Patients with symptomatic brain metastasis (confirmed or suspected);

15) Patients with a history of other malignant tumors in the past 5 years, except for cured skin basal cell carcinoma, cervical carcinoma in situ and superficial bladder cancer;

16) Concomitant diseases that seriously endanger patients' safety or affect patients' completion of this study, as judged by the investigators.

### 3.4.3. Removal Criteria

Subjects who have been included but subject to any of the following circumstances should be removed from the study:

1) The subject is found to be mistakenly included in the trial because he/she did not meet the inclusion/exclusion criteria;

2) The subject fails to follow the drug dosage, administration method and course of treatment specified in this study protocol (but he/she should be observed for drug-related AEs).

### 3.4.4. Withdrawal Criteria

In case of any of the following circumstances, the investigator should discontinue the study and ask the subject to withdraw from the study. Before withdrawal, the investigator should complete the latest evaluation of all indicators, and record in detail the reason and date of the subject's withdrawal.

1) The subject voluntarily withdraws the Informed Consent form and ask to withdraw from the study;

2) The subject has disease progression with medical imaging evidence (e.g., CT, MRI examination results), or clinical progress clearly judged by the investigator (need to record the reasons to judge progress in detail);

3) The subject gets pregnant during the study;

4) The subject delays medication for over 4 weeks;

5) The subject is intolerant to drug toxicity;

6) The subject develops unexpected or unacceptable drug-related adverse reactions;

7) The subject is complicated by other diseases, and is not suitable to continue the clinical study at the investigator's judgment;

8) The subject is poorly compliant, and fails to take drugs on time and at the required dose, or fails to follow the procedures of the study;

9) Other circumstances where the investigator believes it is necessary to ask the subject to withdraw from the study.

### 3.4.5. Protection of Subjects' Rights and Interests

The investigator must state to subjects that their participation in this clinical study is voluntary, and they have the right to withdraw from the study at any stage without being discrimination or retaliation, without their medical treatment or rights or interests being affected, and they can still receive treatment in other ways after withdrawal. The investigator should also make subjects aware that their participation in the study and their personal information in the trial will be kept confidential. Subjects should also be informed of the nature, purpose, expected benefits, and possible risks and inconvenience of the clinical study, optional therapies, as well as their rights and obligations under the *Declaration of Helsinki*, and they should be given sufficient time to decide whether they are willing to participate in the study and asked to sign the Informed Consent form.

## 3.5. Concomitant Treatment

### 3.5.1. Hematology Support

Prophylactic use of colony stimulating factors (G-CSF or GM-CSF) or erythropoietin is prohibited. If a subject developed febrile neutropenia and grade 3 to 4 neutropenia after the first course of treatment after enrollment, or the investigator believes his/her life may be threatened, therapeutic use of colony stimulating factors is allowed. The subsequent courses of treatment can be given corresponding G-CSF support as the disease requires.

### 3.5.2. Treatment of Non-hematological Toxicity Symptoms

Supportive treatment such as the use of antiemetics, antibiotics, analgesics or blood products can be adopted. Oral rinse can be used as therapeutic or prophylactic treatment for stomatitis.

Non-conventional therapies (e.g., herbal medicine or acupuncture) and vitamins/minerals can be used if the investigator believes that they do not affect the outcome of the study. Patients can be given bisphosphonates for osseous metastasis during treatment. Palliative radiotherapy is allowed to irradiate a small area (must be less than 5% of the bone marrow area) if systemic treatment or local pain relief does not effectively control the painful osseous metastasis lesion. All clinical complications and AEs should be positively treated. All drugs used together with Apatinib should be recorded in the CRF in strict accordance with the GCP requirement.

### 3.5.3. Others

Patients are not allowed to receive any other investigational therapies or any other anti-tumor therapies.

## 3.6. Dose Delay and Adjustment

The dose of Apatinib and Capecitabine can be reduced in the study. For Apatinib, the reduction levels are from 500 mg once daily to 500 mg and 250 mg on alternate days, to 250 mg once daily. For capecitabine, 25% and 50% dose reduction will be given. If the subject is still intolerant to the toxicity after the dose is reduced, he/she should be excluded from the study. During the entire study period, the dose is allowed to be reduced at most twice, and shall not be increased.

The drug can only be suspended or its dose can only be reduced when a subject has grade III or worse adverse events or grade II intolerable toxicity assessed by the clinicians. Controllable nausea, vomiting and fever (below 38°C) with a clear cause can be actively handled and treated symptomatically without suspending the drug or reducing its dose. The dose and suspension of the drug should be stated in the final study report.

To ensure the drug intensity to the subjects in the trial, the time of each drug suspension and the cumulative time of suspension during each administration cycle shall not be over 14 days, and the suspension shall not exceed two times during each cycle. If the drug discontinuation time or the number of suspensions exceeds the requirement, the subject will withdraw from the study, but his/her tumor shall still be evaluated following the study procedure.

## 3.7. Symptomatic Treatment of Common Adverse Reactions of Apatinib and Capecitabine

### 3.7.1. Hypertension

Tumor patients with hypertension should use anti-hypertension drugs appropriately to control their blood pressure before receiving anti-VEGF/VEGFR drugs, while tumor patients with poorly controlled hypertension should use anti-VEGF/VEGFR drugs with caution. Early blood pressure control is important for protecting the renal function. The elevated blood pressure in those patients during treatment will drop after the discontinuity of drug, and generally no further treatment is needed. However, patients with significantly elevated blood pressure (blood pressure ≥ 140/100 mmHg) and/or corresponding symptoms should be given antihypertensive treatment. Current routine combinations of drugs for hypertension are the combined use of ACEIs or ARBs with diuretics, calcium channel blockers with β-receptor blockers, ACEIs with calcium channel blockers, diuretics with β-receptor blockers, and α-receptor blockers with β-receptor blockers. The common antihypertension drugs, including Diuretics, β-receptor blockers, Calcium channel blockers, Angiotensin converting enzyme inhibitors, Angiotensin II receptor blockers should be considered in clinical practice.

**Grading criteria and prevention and treatment recommendations for hypertension (NCI-CTCAE 4.0)**

| Grading | Definition | Prevention and treatment recommendations |
| --- | --- | --- |
| Grade 1 | Systolic pressure of 120 to 139 mmHg  Or diastolic pressure of 80 to 89 mmHg | Closely monitor the blood pressure; limit salt intake, and quit smoking and alcohol;  Continue to use Apatinib without dose adjustment |
| Grade 2 | Systolic pressure of 140 to 159 mmHg  Or diastolic pressure of 90 to 99 mmHg | Closely monitor the blood pressure; continue to use Apatinib without dose adjustment in normal cases; Should take anti-hypertension drugs and should not discontinue anti-hypertension drugs at will |
| Grade 3 | Systolic pressure ≥ 160 mmHg Or diastolic pressure ≥ 100 mmHg | Suspend Apatinib; Single-agent poorly controlled hypertension, combined medication should be considered;; consult and receive treatment from cardiovascular specialists; closely monitor the blood pressure; reduce the dose of Apatinib and continue to use it if the blood pressure is well controlled |
| Grade 4 | Life-threatening (malignant hypertension or persistent nerve injury, hypertensive crises) | Discontinue Apatinib immediately and permanently; consult cardiovascular specialists for active treatment of hypertension, and closely monitor the blood pressure and other vital signs |
| Grade 5 | Death |  |

Note: Patients with hypertensive crisis should discontinue Apatinib permanently and withdraw from this clinical study.

### 3.7.2. Proteinuria

For all cancer patients treated with anti-VEGF/VEGFR drugs, urine protein must be closely monitored during treatment, especially during the first 6 weeks of treatment. It is recommended to check urine routine and/or 24-hour urine protein quantification every 2 weeks for the first 2 months, and then check every 4 weeks. Patients with positive results of urine protein should have creatinine test on a regular basis to assess renal function. Patients with urine protein +++ or 24-hour urine protein > 3.5 g should consult with a nephrologist and consider stopping the drug appropriately. Patients with stage 3 chronic kidney disease or above should take apatinib with caution. As ACET and ARB drugs may reduce the pressure in the kidney tubules, thus reducing proteinuria, they can be taken as appropriate.

**Apatinib-related proteinuria classification standard (NCI-CTCAE4.0) and recommendations for prevention and treatment**

| Grading | Definition | Prevention and treatment recommendations |
| --- | --- | --- |
| Grade 1 | Urine protein (+)  Or 24-hour urine protein quantification < 1.0g | Continue to take apatinib without dose adjustment;  Pay attention to observation |
| Grade 2 | Urine protein (++)  Or 24-hour urine protein quantification: 1.0-3.4g | a. Continue to take apatinib, generally no dose adjustment required;  b. Drug intervention should be considered;  c. Monitor 24-hour urine routine and 24-hour urine protein quantification |
| Grade 3 | 24-hour urine protein quantification ≥ 3.5g | a. Suspend taking Apatinib;  b. Consult with a specialist in nephrology;  c. Conduct drug intervention;  d. After proteinuria returns to ≤2 grade, the dosage of Apatinib can be reduced;  e. If grade 3 proteinuria still occurs after 2 dose reductions, the Apatinib treatment shall be permanently terminated |

### 3.7.3. Hand-Foot Skin Reaction

**HFSR classification standard (NCI-CTCAE 4.0)**

| Grading | Definition |
| --- | --- |
| Grade 1 | Minor skin changes or dermatitis (local erythema, edema, hyperkeratosis, painless) without affecting daily life |
| Grade 2 | Obvious skin changes (flaking, blisters, bleeding, swelling, hyperkeratosis), pain, affecting daily life and activities |
| Grade 3 | Severe skin changes (flaking, blisters, ulcers, bleeding, edema, hyperkeratosis), obvious pain, with limited personal self-care ability |

Suggestions: Early prevention of HFSR can lower the severity of adverse reactions. Therefore, from the first day of taking Apatinib, do not wait until the HFSR occurs, the following is recommended:

1. Patients are recommended to avoid squeezing their hands and feet, to wear loose and soft shoes, and to avoid stimulation by excessive cold and heat;
2. Patients are recommended to apply urea ointment / moisturizing cream or aloe vera gel to the hands and feet on the first day of taking Apatinib.
3. If HFSR has occurred, skin care shall be strengthened to keep the skin clean to avoid infection. When the skin is not damaged, magnesium sulfate solution (1-3%) or honeysuckle of 50 g may be used to soak hands and feet in water for 10-15 minutes each time, the moisture can be absorbed with a soft towel, then urea ointment can be applied for moisturizing.
4. B vitamins can be taken appropriately. If it is difficult to relieve the pain, Celecoxib can be taken.
5. When intolerable pain occurs, it is recommended to stop taking Apatinib, and wait for 2 to 3 days after stopping the drug, and resume the drug after the pain is relieved.. Recovering medication after stopping will generally reduce the degree of pain.

### 3.7.4. Skin Rash

Skin rash usually appears about 1 week after medication. It is most obvious in the dorsal joints of the limbs, such as the elbow, wrist, knee, and ankle joints. It may manifest as macule, papules, pruritus, desquamation and ulcers, most of which will disappear within 1 to 2 weeks after appearance.

Treatment method: a. External use: Compound Dexamethasone Acetate, Eloson, calamine lotion, mupirocin ointment; b. Oral: anti-allergic drug such as Clarityne

### 3.7.5. Bleeding

Patients using the drug should be closely monitored for coagulation function, and in case of severe abnormal coagulation function (grade 3 to 4), drug suspension is suggested. If massive gastrointestinal bleeding occurs, Apatinib must be discontinued immediately, and bleeding should be treated actively based on conventional clinical practice.

### 3.7.6. Mouth Ulcers

Mouth ulcers caused by targeted drugs are usually discrete, ovoid, shallow, with clear boundaries and red surroundings. But some patients develop oral pain, dysgeusiae and swallowing difficulty rather than visible ulcers. Its pathogenesis has not yet been defined. Studies have shown that it is related to cell-mediated immunotoxicity and immune complex formation. It is also related to the declined VEGF level in the saliva. According to the literature, the incidence of mouth ulcers in patients using Sunitinib and Regorafenib was 25–38%, generally mild to moderate.

Apatinib and Capecitabine often cause mild to moderate mouth ulcers that can be controlled by symptomatic treatment. Few patients have severe or long-lasting mouth ulcers, in this case, the dose should be reduced or the treatment should be discontinued. It is recommended to educate patients to maintain oral hygiene and avoid eating hard, cold, hot or spicy food. Additionally, oral vitamin B can relieve mouth ulcers to a certain extent.

In the event of mild mouth ulcers, Yunnan Baiyao, Kangfuxin oral liquid, Yinlian gargle, Chlorhexidine oral ulcer mucous patch, or Beifuji spray, GeneTime spray, as well as Bing Peng San, Qing Dai San and Yangyin Shengji San are the preferred drugs. Patients with obvious oral pain can be topically added 2% Lidocaine and Sucralfate. For severe cases, corticosteroids or antibiotics can be used topically.

### 3.7.7. Bone Marrow Suppression

Antibiotics should be used for infection prevention when the neutrophil count was less than 0.5 × 10^9^/L, and patients with fever and coinfection should be treated with broad-spectrum antibiotics, such as granulocyte colony stimulating factors (G-CSFs).

In the event of transient thrombocytopenia (platelet count less than 50 × 10^9^/L), a small dose of glucocorticoid or Etamsylate can be used to prevent bleeding. When the platelet count is less than 20 × 10^9^/L, platelet transfusion, Etamsylate and hormones (e.g., Prednisolone) should be considered, as well as thrombopoietin (TPO) or interleukin to simulate the growth and differentiation of megakaryocytes when necessary.

### 3.7.8. Nausea, Vomiting and Loss of Appetite

Nausea and vomiting are common adverse events in patients with advanced tumors. Patients with mild to moderate symptoms can be given Metoclopramide/Metaline, while patients with severe symptoms need to be treated with 5-HT3 receptor blockers (Ondansetron, Granisetron, etc.), and patients with severe dehydration should be appropriately supplemented with fluids and electrolytes.

### 3.7.9. Hepatic Function Impairment

During treatment, patients should be regularly monitored for the hepatic function status. It is recommended to check liver function once every two weeks during the first two months of medication. Patients with severe hepatic function insufficiency are not allowed to use Apatinib and Capecitabine. For patients with elevated grade 3/4 transaminase and total bilirubin, it is recommended to suspend the drug, strengthen the symptomatic treatment to protect the liver and reduce the enzyme. Monitor the patient’s transaminase and total bilirubin levels and restore medication after such levels significantly drop, and also reduce the dose if the patient develops grade 3/4 adverse events again after restoration of medication.

## 3.8. Study Procedures

All patients will go through three stages of the screening period, treatment period and end of treatment period to complete this clinical study after signing the Informed Consent form. Before the study begins, all patients must read and sign the Informed Consent form approved by the Ethics Committee (EC). All study steps need to be performed within the time window specified in the trial flow chart. All observation indicators and time of examinations should not be affected by the length of drug discontinuity, and relevant examinations should be carried out in each corresponding course of treatment according to regulations.

### 3.8.1. Screening Stage

**1) Unless otherwise specified, the following screening procedures must be completed within 4 weeks before the study drug treatment starts:**

• Sign the Informed Consent form;

• Collect demographic data: Gender, date of birth, nationality, height, weight, etc.;

• Tumor diagnosis: Date of diagnosis, histological typing, lesion (primary or metastatic lesion), pathological stage TNM, clinical stage;

• Tumor treatment history:

a. Tumor surgery: Date and name of surgery

b. Radiotherapy history: Time and dose of radiotherapy received

c. Chemotherapy history and targeted treatment history: Name of the drug used, dose, medication cycle, start and end time, reason for dressing change (for dressing change due to disease progression, the imaging evidence should be recorded; for changing of therapies due to intolerance, adverse events and severity should be recorded), efficacy and outcome;

• Date of disease progress, relapse or metastasis after the last treatment;

• Complications treatment history: Including the treatment history of diabetes, hypertension, COPD, etc.;

• Blood pressure monitoring: During the screening period, the investigator will monitor the blood pressure of subjects. Before each blood pressure measurement, subjects are required to avoid smoking and coffee for 30 minutes and rest quietly for at least 10 minutes. During the measurement, they are required to be seated and maintain the elbow and the heart at the same level. Each blood pressure measurement is taken ipsilaterally.

• Imaging examination: Head, chest and abdominal CT or MRI. Bone scan is required when bone metastasis is clinically suspected;

(Written Informed Consent form must be obtained before any medical operation specified in the study. But CT/MRI scanning results obtained before signing the Informed Consent form to participate in this trial can be used for tumor evaluation during the screening period as long as they meet the requirements, provided that the date of such CT/MRI scanning must be within 28 days before the treatment)

• UCG: LVEF will be observed carefully; (Results of ECG examinations performed within 28 days before the signature of the Informed Consent form are acceptable)

• Concomitant medication and treatment: Concomitant medication and treatment within 28 days before enrollment and during the study period will be recorded.

• Record adverse events: From the date of signing the Informed Consent form, adverse events will be recorded regardless of whether the patient uses drugs or not.

**2) The following screening procedures must be completed within 7 days before the study drug treatment starts:**

• ECOG scoring;

• Vital signs: Heart rate, respiratory frequency, body temperature, blood pressure;

• Physical examination: Head and face, skin system, lymph nodes, eyes, ear, nose and throat, mouth, respiratory system, cardiovascular system, abdomen, genitourinary system, musculoskeletal system, nervous system and mental state;

• Blood routine: Hemoglobin, red blood cells, leukocytes, neutrophils, lymphocytes, platelets;

• Urine routine: Urine protein, urine sugar, urine occult blood (urine red blood cell, white blood cell), urine pH and urine ketone bodies;

If the semi-quantitative test shows protein ≥ 2+, the 24-hour urine protein quantitative test should be performed. All patients included must have a 24-hour urine protein level of less than 1g.

• Fecal routine: Feces OB test;

• Blood biochemistry test: Total bilirubin, direct bilirubin, indirect bilirubin, ALT, AST, AKP, r-GT, total protein, albumin, urea nitrogen, creatinine, uric acid, blood glucose, triglyceride, cholesterol, blood lipase, blood amylase, potassium, sodium, chlorine, calcium and phosphorus;

• Coagulation function test: PT, APTT, TT, Fbg and INR;

• EB virus detection (by PCR);

• Blood sample: If a patient is to be included certainly, his/her peripheral blood should be collected to determine serum VEGF-A/C and VEGFR-2 levels, and VEGF and VEGFR2 gene polymorphisms;

• ECG: 12-lead ECG examination. QT, QTc and P-R intervals will be observed carefully, and ECG will be performed for three consecutive times to identify abnormalities (with a 5-minute interval each time and the QTc interval marked);

• Myocardial enzyme spectrum examination: Patients will only receive one examination within 7 days before the inclusion, no examination is needed in the future, and supplementary examination is only performed when the ECG is abnormal;

• Pregnancy test (for female patients of child-bearing age);

### 3.8.2. Treatment Stage

All subjects will proceed to the treatment period if successfully included in the screening period, with 21 days as a treatment cycle. Observational visit will be conducted on Day 21 ± 3 of the first cycle, and on Day 21 ± 3 of each cycle starting from the second cycle. Imaging evaluation will be performed every two treatment cycles until subject has disease progression or withdraws due to intolerant toxicity.

• ECOG scoring: Once at the end of each cycle;

• Physical examination;

• Blood routine: Hemoglobin, red blood cells, leukocytes, neutrophils, lymphocytes, platelets, once at the end of each cycle;

• Urine routine: Urine protein, urine sugar, urine occult blood (urine red blood cell, white blood cell), urine pH and urine ketone bodies; If the semi-quantitative test shows protein ≥ 2+, the 24-hour urine protein quantitative test should be performed, once at the end of each cycle;

• Blood biochemistry test: Total bilirubin, direct bilirubin, indirect bilirubin, ALT, AST, AKP, r-GT, total protein, albumin, urea nitrogen, creatinine, uric acid, blood glucose, triglyceride, cholesterol, lipase, blood amylase, potassium, sodium, chlorine, calcium and phosphorus. Once at the end of each cycle;

• Coagulation function test: PT, APTT, TT, Fbg and INR, once at the end of each cycle;

• ECG: 12-lead ECG examination. QT, QTc and P-R intervals will be carefully observed. In case of precordial pain or palpitations, the ECG should be checked immediately, and the myocardial enzyme spectrometrum examination should be added at the same time. In case of abnormal ECG findings with significant clinical significance, supplementary UCG check should be performed. Once at the end of each cycle;

• Imaging examination:

a. The target lesion confirmed at baseline should be checked again under the same conditions of the baseline examination (the same thickness of the scanning layer and the same use of the contrast agent). For patients found with osseous metastasis at baseline, the femoral lesion should be checked again. Other lesions found at baseline and new suspected lesions in later period should also be checked as appropriate;

b. In the event of suspected disease progression (e.g., deterioration of symptoms) or when PR or CR evaluation needs to be confirmed (three weeks after the evaluation), extra unplanned imaging examination can be performed.

• Adverse events: Adverse events should be recorded from the first medication of the study drug till at least 30 days after the last medication, and should be followed up until such adverse events are relieved or turn stable;

• Combined medication: Concomitant medication and concomitant treatment during the study should be recorded. Once the subject discontinue the treatment of the study, only the concomitant medication and concomitant treatment for new or unresolved treatment-related adverse events should be recorded;

• Study drug: Distribution and recycling of the study drug should be recorded once at the end of each cycle.

### 3.8.3. End of Treatment/Withdrawal from the Study

Upon the end of the study treatment or withdrawal from the study, if the subject has not undergone any examination within 14 days before the study treatment ends, the following examinations should be performed:

• ECOG scoring;

• Vital signs: Heart rate, respiratory frequency, body temperature, blood pressure;

• Blood pressure examination: Investigators will measure the blood pressure of the subjects. Before the measurement, subjects are required to avoid smoking and coffee for 30 minutes and rest quietly for at least 10 minutes. During the measurement, they are required to be seated and maintain the elbow and the heart at the same level. Each blood pressure measurement should be taken ipsilaterally;

• Physical examination: Head and face, skin system, lymph nodes, eyes, ear, nose and throat, mouth, respiratory system, cardiovascular system, abdomen, genito-urinary system, musculoskeletal system, nervous system and mental state;

• Blood routine: Hemoglobin, red blood cells, leukocytes, neutrophils, lymphocytes, platelets;

• Urine routine: Urine protein, urine sugar, urine occult blood. If the semi-quantitative test shows protein ≥ 2+, the 24-hour urine protein quantitative test must be performed;

• Blood biochemistry test: Total bilirubin, direct bilirubin, indirect bilirubin, ALT, AST, AKP, r-GT, total protein, albumin, urea nitrogen, creatinine, uric acid, blood glucose, triglyceride, cholesterol, lipase, blood amylase, potassium, sodium, chlorine, calcium and phosphorus;

• Coagulation function test: PT, APTT, TT, Fbg and INR;

• ECG: 12-lead ECG examination;

• UCG;

• Imaging examination: If a subject has not undergone any imaging examination within 4 weeks before the treatment ends, he/she should undergo one imaging examination upon the end of treatment or withdrawal from the study. For patients with non-imaging evidence of tumor progression (intolerance or other conditions), tumor evaluations should be performed once every two cycles until disease progression, death or the start of other anti-tumor therapies;

• Pregnancy test: Female patients of child-bearing age;

• Adverse events evaluation;

• Recycling of the study drug.

### 3.8.4. 30-day Follow-up after Subject Withdrawal

All subjects should continue to perform safety evaluation and adverse event follow-up within 30 days after the end of the last medication, with their concomitant treatment recorded.

• Record concomitant medication/treatment;

• Adverse events follow-up.

### 3.8.5. Survival Follow-up

After the 30-day follow-up after the discontinuity of medication, the subject proceeds to the survival follow-up period. At least once every 3 months, the subject, his/her family member or local doctors were followed up by phone calls to collect survival data (date and cause of death) and information after the end of the study treatment (including treatments received afterwards) until death, loss of contact or the sponsor terminates the study. Each survival follow-up should be recorded in the follow-up table in detail.

The subject may have adverse events during the trial and requires unplanned follow-up, in which the following items should be recorded:

• Record concomitant medication/ treatment;

• Record adverse events in the follow-up;

• Record all relevant examinations received (including imaging examination).

## 3.9. Efficacy Evaluation

The efficacy is determined in strict accordance with the RECIST 1.1 criteria.

### 3.9.1. Measuring Method

For both pre-treatment baseline measurement and efficacy evaluation, the same techniques and methods should be used to evaluate the lesion, and such measuring methods should be easily repeatable and enable easy data storage, such as CT and MRI.

Contrast-enhanced CT is the preferred method to evaluate tumors. If the patient cannot undergo contrast-enhanced CT, MRI should be performed. For the head, chest, abdomen and pelvis, CT and MRI scanning should be performed with 10 mm section thickness or thinner, and spiral CT scanning should be performed continuously with 5 mm section thickness.

For superficial lesions that are clinically measurable (e.g., skin nodules and palpable lymph nodes), color images containing a scale to indicate the size of lesions are recommended for the estimation of the lesion size.

Ultrasound should not be used to measure tumor lesions that are clinically difficult to measure (e.g., visceral lesions) in overall response evaluation. Ultrasonography can be used as an optional method for clinical evaluation of superficial palpable lymph nodes, subcutaneous nodules and thyroid nodules. Ultrasonography can also help to confirm the complete disappearance of superficial lesions that are usually evaluated by clinical examination.

### 3.9.2. Record Tumor Measurement Data

Target lesions should include all the organs involved, with a maximum of 5 target lesions in each organ, and the total number of all target lesions should not exceed 10. They should be measured and recorded at baseline and at the time points specified. Target lesions should be selected based on the size (lesions with the largest diameter) and suitability for repeated accurate measurability (with imaging technique or clinical methods). A primary lesion should be classified as a target lesion only if it can be accurately measured.

The longest diameter of each target lesion will be recorded. The sum of the longest diameters of all target lesions will be calculated and recorded. During the treatment, the objective response of tumors in measurable dimensions will be further clarified by referring to the sum of the longest diameters at baseline.

All non-measurable lesions and other measurable lesions not selected as target lesions should be identified as non-target lesions, and should also be recorded at baseline. Measured data of such lesions are not required in the study, but their presence and disappearance should be tracked throughout the study period.

### 3.9.3. Efficacy Evaluation Indicators

#### 3.9.3.1. Primary Efficacy Indicators

Objective response rate (ORR): The proportion of patients whose tumors shrink to a certain size and maintain such size for a certain period of time, including patients with complete response (CR) and partial response (PR).

The RECIST 1.1 criteria for solid tumor response will be used to evaluate the objective response of tumors. Subjects should have measurable tumor lesions at baseline, and the efficacy evaluation criteria will be divided into CR, PR, SD and progressive disease (PD) according to the RECIST 1.1 criteria.

#### 3.9.3.2. Secondary Efficacy Indicators

Disease control rate (DCR): The proportion of patients with response and stable disease (SD) after treatment, including patients with CR, PR and SD.

Duration of Response (DoR): Duration from the first PR to PD.

Progression-free survival (PFS): Duration from the date of inclusion of the patient to the date of any recorded tumor progression or death from whatever cause. Analysis of this indicator includes tumor evaluation results during treatment and the follow-up period. If a patient has several indicators that can be judged as PD, the indicator that appears first should be used for PFS analysis. Relapse, new lesion or death will be considered as the outcome of the study. If the patient is receiving other systemic anti-tumor treatment or treatments for the observed target lesions, he/she should also be regarded as PD. For a patient who has no PD or dies when the study ends, the last time he/she does not have PD will be taken as the censored data.

Overall survival (OS): Duration from the date of inclusion to the date of death from whatever cause. For a subject still alive at the last follow-up, his/her OS is counted as data censored at the last follow-up time. OS for data censoring is defined as the duration from the inclusion dateto the censoring date.

### 3.9.4. Safety Evaluation

**3.9.4.1. Adverse Event (AE) Evaluation**

Definition of AE: AE refers to any adverse medical event that occurs in the subject or clinical trial subject after receiving a drug or treatment therapy, but not necessarily causally related to treatment.

An AE can be any unpleasant or unrelated sign (including abnormal laboratory findings), symptom or illness that are time-related to the the use of the medical product, whether or not it is considered to be related to the medical product.

Events that occur during pre-treatment and post-treatment periods are also considered AEs according to regulations. Therefore, safety monitoring reports on AEs or serious adverse events (SAEs) should start from the date on which subjects are entering the trial (sign the Informed Consent form) till the date of the end of the trial follow-up.

**3.9.4.2. Record AEs**

The names, severity, time of occurrence, duration, treatment measures and outcomes of all AEs during the trial should be recorded in detail, and filled in truthfully in the case report form (CRF). Abnormal laboratory examination data should be recorded in the CRF, and the examination should be repeated at least once a week, and the abnormal data should be followed up till normal or the study ends. All AEs that occur within 30 days after the end of the last medication should be reported and recorded.

**3.9.4.3. Judgment of The Relationship between AEs and The Study Drug**

Possible relationships between AE and the study drug will be evaluated as " definitely related, probably related, possibly related, unlikely relatedt and not related" using the five-tier classification method (see the table below). AEs with a relationship of the first three tiers are judged to be related to the study drug. When calculating the incidence of adverse reactions, the total number of patients in the first three tiers should be taken as the numerator, and the total number of all subjects participating in safety evaluation should be taken as the denominator.

**Criteria for judging the relationship between AEs and the study drug**

| Criteria | Definitely related | Probably related | Possibly related | Unlikely related | Not related |
| --- | --- | --- | --- | --- | --- |
| The time sequence is reasonable | Yes | Yes | Yes | Yes | No |
| The drug reaction type is known | Yes | Yes | Yes | No | No |
| The removal cause can be improved | Yes | Yes | Yes or no | Yes or no | No |
| It will occur again upon next medication | Yes | ? | ? | ? | No |
| The reaction may have another explanation | No | No | No | Yes | Yes |

### 3.9.5. Serious Adverse Event (SAE)

**3.9.5.1. Definition of SAE**

SAE refers to medical events occurring during the clinical trial that requires hospitalization or prolonged hospitalization, makes thepatient disabled, affects the ability to work, endangers life or leads to death, or causes congenital malformations. It includes the following unexpected medical events:

• Events leading to death;

• Life-threatening events (defined as the risk of death of the subject when the event occurs);

• Events requiring hospitalization or prolonged hospitalization;

• Events that can lead to permanent or severe disability/ dysfunction;

• Carcinogenic or teratogenic events;

• Drug overdose.

**3.9.5.2. Hospitalization**

AEs resulting in hospitalization or prolonged hospitalization in clinical studies should be regarded as SAEs. All patients that are admitted to medical institutions for the first time (even if for less than 24 hours) meet this standard.

The following circumstances should not be regarded as hospitalization:

• The patient is admitted to a rehabilitation facility

• The patient is admitted to a sanatorium

• The patient is routinely admitted to the emergency room

• The patient undergoes an operation on the date of hospitalization (such as outpatient/same day/ambulatory surgery)

Hospitalization or prolonged hospitalization irrelevant to the deterioration of AEs should not be seen as SAEs, such as:

• The patient is admitted to the hospital for pre-existing disease and develops no new adverse events and has no deterioration of the pre-existing disease (e.g., the persisted laboratory abnormalities found before the study);

• Hospitalization for management reasons (e.g., annual routine physical examinations);

• Hospitalization specified in the study protocol during the clinical trial (e.g., operations specified in the study protocol);

• Elective hospitalization irrelevant to the deterioration of AEs (e.g., elective cosmetic surgeries);

• Scheduled treatment or surgeries should be recorded in the entire trial protocol and/or in the subject's baseline information;

• Hospitalization only for the use of blood products;

Diagnostic or therapeutic invasive (e.g., surgeries) and non-invasive procedures should not be reported as AEs. However, if the disease condition causing such an operation meets the definition of AE, it should be reported. For example, acute appendicitis developed during the reporting period of AEs should be reported as AE, and the appendectomy thus performed should be reported as the treatment of the AE.

**3.9.5.3. Drug Overdose**

Drug overdose refers to the increase of dose of the study drug to the subject within 24 hours (depending on the specific protocol), exceeding the dose prescribed by the investigator. All cases of the study drug overdose, whether related to AEs/SAEs or not, should be reported as SAEs.

**3.9.5.4. SAE Reporting Procedures**

SAEs should be reported from the date on which a subject signs the Informed Consent form till 30 calendar days (including the 30th day) after the last medication of the study drug. During the study period, all SAEs should be reported to clinical monitors and principal investigator within 24 hours after occurring, and recorded in the signed and dated *Serious Adverse Event (SAE) Report Form on Clinical Study of New Drug*, and faxed immediately to the sponsor, group leader unit, the ethics committee of the research unit, the CFDA and the food and drug administration of the region (province or city) where the investigator is located.

All SAEs that occur during the continuity of drug supply after the study ends should be reported to the sponsor within 24 hours. The information of all SAEs should be recorded in the SAE report form. All SAEs that occur during continuity of drug supply and within 30 days after the last medication should be reported. SAEs that occur 30 days after the last medication are not necessarily reported unless they are suspected to be related to the study drug.

All SAEs should be recorded in detail the symptoms, severity, time of occurrence, time of treatment, measures taken, time and manner of follow-up, and outcomes. When a SAE is believed irrelevant to the study drug and possibly related to the study conditions (e.g., termination of original treatment or complications during the study) by the investigator, such relationship should be described in detail in the SAE page in the medical record form. If the severity of an ongoing SAE or its relationship with the study drug changed, the SAE follow-up report should be submitted to the sponsor immediately. All SAEs should be followed up till they disappear or stabilize.

**SAE Contact**

| Unit | Contact | Telephone | Fax |
| --- | --- | --- | --- |
| National Medical Products Administration (NMPA) | Safety Supervision Division | 010-68313344-1003 | 010-88363228 |
| Sun Yat-sen University Cancer Center | Ethics Committee | 020-87343535 |  |
| Jiangsu Hengrui Medicine Co., Ltd. | Medical Services System | 021-68868570 | 021-50819731 |

## 3.10. Ethical, Regulations and Administrative Principles

### 3.10.1. Ethical Principles

The study will be conducted in accordance with the principles established by the 18th World Medical Association Congress (Helsinki, 1964) and all subsequent amendments.

### 3.10.2. Informed Consent

It is the responsibility of the investigator or his/her representatives (if permitted by local law) to explain in detail to each subject participating in the study of the purpose, methods, benefits and potential risks of the study, and to obtain the written Informed Consent form signed by each subject. Subjects who are unable to sign the Informed Consent form should have it signed by their legal representatives. If neither the subject nor the legal representative can read, a notary should be present throughout the informing process. After the subject and the legal representative agree orally to participate in the study, the notary should sign the Informed Consent form and state that its contents have been accurately explained and understood. Investigators or their representatives should also state that the subjects have the full right to refuse to participate in the study or discontinue the study at any time. The case report of the study is attached with the Informed Consent form of the subjects, which must be completely filled in. If new safety information changes the risks/benefits evaluation, the contents of the Informed Consent form may be modified/updated as necessary. Upon such modification/update, all subjects (including those receiving chemotherapy) should be notified and required to sign the modified Informed Consent form for their consent to continue participating in the study.

### 3.10.3. Data Protection

Personal information of patients and the investigator may be included in the database of Sun Yat-Sen University Cancer Center, and should be processed conforming to all applicable local laws and regulations. When archiving or processing investigator-related and/or patient-related personal information, Sun Yat-Sen University Cancer Center will take all appropriate measures to protect and prevent any unauthorized third party from accessing such data.

### 3.10.4. Confidential Agreement

All materials, data (oral or written) and unpublished documents provided to the investigator (or any action taken by the sponsor on behalf of the investigator), including this protocol and the CRF, should be the exclusive property of Sun Yat-Sen University Cancer Center. Without the prior official written consent of Sun Yat-Sen University Cancer Center, the investigator or any member of his/her group is not allowed to disclose such materials or information to any unauthorized person. The investigator shall keep confidential all the information received, obtained or inferred during the course of the study, and shall take all necessary measures to ensure that no disclosure is made, except as permitted by law.

### 3.10.5. Record Keeping

The investigator shall arrange the keeping of the study documents till the end of the study. Besides, the investigator shall adhere to specific local regulations/guidelines regarding the patients' records keeping. Unless otherwise stated in the investigator agreement, in accordance with other standards and/or local laws, it is recommended that the investigator save the study documents for at least five years after the completion or suspension of the study.

### 3.10.6. Early Suspension of The Study

Sun Yat-Sen University Cancer Center can decide to suspend the study at any time for any reason, which shall be communicated to the participating investigator in writing. Similarly, if the investigator decides to withdraw from the study, he/she shall notify Sun Yat-Sen University Cancer Center in writing. If applicable, the ethics committee (IRB) and the health regulatory authority shall be notified in accordance with local regulations.

### 3.10.7. Inspection by Sponsor and The Regulatory Authority

The investigator agrees to provide direct access to the study records for review by the inspectors assigned by the sponsor/regulatory authority, and understands that such personnel are subject to the occupational obligation of confidentiality and will not disclose any personal identity or personal medical information of patients. The investigator will make every effort to assist in such inspections and to allow inspectors to have access to all necessary equipment, data and documents. The confidentiality of verification data and the protection of patients should be respected during such inspections. The investigator shall immediately communicate with the sponsor the results and information given by the regulatory authority after the inspections. The investigator shall take appropriate measures according to the requirements of the sponsor, and shall correct all problems found in the inspections.

## 3.11. Data Collection and Management

### 3.11.1. Data Entry and Modification

Data should be input and managed by an independent data management unit. Data administrators should use EpiData 2.0 to compile data data entryprogram for data entry and management. To ensure the accuracy of data, data should be input in duplicate independently and proofread by two data administrators. The questions in the case report form should be written in the Doubt Report Questionnaire (DRQ) by data administrators and then sent by clinical monitors to the investigator. The investigator should reply and send back the questionnaire as soon as possible, so that the data administrators can modify, confirm and enter the data according to the investigator's replies. When necessary, the DRQ can be sent again.

### 3.11.2. Data Lock

After data are reviewed and the established database is confirmed to be correct, the principal investigator, sponsor and statistical analysts should lock the data. The locked data file will no longer be modified.

### 3.11.3. Statistical analysis Data Set

Full analysis set: It covers all patients included in the study who have used the drug at least once for efficacy analysis based on the intention-to-treat (ITT) principle. For the case data for which the entire treatment process cannot be observed, the last observation data should be carried forward to the final outcome of the trial.

Per-protocol set: It covers all patients who conform to the study protocol, have good compliance, have not taken any prohibited drugs during the study period and complete the case reports as required. No imputation will be made on the missing data. FAS and PPS are statistically analyzed for the efficacy of the drug.

Safety analysis set: It covers all patients included in the study who have used the study drug at least once, and have safety records after medication. This dataset is used for safety analysis.

### 3.11.4. Statistical Analysis Plan

The study results are mainly analyzed by descriptive statistics. The measurement data will be expressed as mean, standard deviation, median and maximum and minimum values, and the enumeration data and ranked data will be presented as frequency (composition ratio), rate and confidence interval.

The two-tailed test will be used for all statistical tests. When the P-value is 0.05 or lower, the difference will be considered statistically significant. The confidence interval adopts 95% confidence level.

1) Basic characteristics of patients

2) Efficacy analysis

The corresponding 95% CIs of ORR and DCR are calculated based on the Clopper-Pearson exact method. The Kaplan-Meier method will be used to estimate the median of the DOR, PFS and OS, and to draw the survival curve.

3) Safety evaluation

Safety evaluation is mainly based on descriptive statistical analyses, and lists the adverse events and adverse reactions occurring in this trial (adverse reactions are defined as adverse events that are 'definitely related/probably related/possibly related to' the test drugs"). The laboratory examination results describe the circumstances that are normal before the trial but abnormal after the treatment, as well as their relationships with the test drugs when abnormal changes occur.

### 3.11.5. Quality Control and Quality Assurance

• The clinical study unit must be a clinical pharmacological research centre eligible for clinical studies recognized by the CFDA.

• Research staff should be physicians trained on clinical trials, and they should work under the guidance of senior professionals;

• Clinical wards should be checked to meet the standardized requirements before the trial to ensure well-equipped rescue;

• It is recommended that professional nursing staff administer the drugs to the subjects, and these staff should have a detailed understanding of the use of the drugs and ensure the compliance of the subjects.

• The research center should conduct the study in strict accordance with the study protocol, and truthfully fill in the case reports;

• An independent imaging evaluation committee should be established. The results of imaging examinations (including MRI and CT) of all patients should be confirmed by the independent imaging evaluation committee. All patients with CR, PR, SD and PD efficacy evaluations must first be reviewed by the person in charge of the research unit (PI), and all imaging data for efficacy evaluation (including image films or imaging examination CDs) must be retained and finally confirmed by the independent imaging evaluation committee. The data required by the independent imaging evaluation committee should be the imaging data of each center, and should be provided by local monitors in the form of CDs.

• Monitors should also supervise the the progress of clinical trial following standard operating procedures, and ensure that all data are accurately and completely recorded and reported, all case report forms are entered properly and consistent with the original data, and the study is conducted conforming to the clinical study protocol.

• In case of an SAE, the research units must be notified in time, and when necessary, the study must be temporarily suspended;

• Each research unit participating in the trial should be inspected by the sponsor and the drug administration department, and more importantly, the investigator and relevant personnel should facilitate such supervision and inspection.

# References

[1]Su SF, Han F,Zhao C,et al.Treatment outcomes for different subgroups of nasopharyngeal carcinoma patients treated with intensity-modulated radiation therapy[J].Chin J Cancer,2011,30(8):565-573.

[2] Lu TX, Han F, Li JX. Advances in clinical studies on recurrent nasopharyngeal carcinoma [J]. China Oncology, 2008, 18(9): 661-665.

[3]Chen C,Wang FH,An X,et al.Triplet combination with paclitaxel,cisplatin and 5-FU is effective in metastatic and/or recurrent nasopharyngealcarcinoma.Cancer Chemother Pharmacol,2013, 71(2):371-8.

[4]Long GX,Lin JW,Liu DB,et al.Single-arm,multi-centre phase II study of lobaplatin combined with docetaxel for recurrent and metastaticnasopharyngeal carcinoma patients.Oral Oncol,2014, 50(8):717-20.

[5]Li Zhang, Yan Huang, Shaodong Hong, et al.Gemcitabine plus cisplatin versus fluorouracil plus cisplatin in recurrent or metastatic nasopharyngeal carcinoma:a multicentre, randomised, open-label, phase 3 trial[J].Lancet 2016,388:1883–1892.

[6]Peng PJ,Ou XQ,Chen ZB,et al.Multicenter phase II study of capecitabine combined with nedaplatin for recurrent and metastatic nasopharyngealcarcinoma patients after failure of cisplatin-based chemotherapy[J].Cancer Chemother Pharmacol, 2013, 72(2):323-8.

[7]Chua D,Wei WI,Sham JS, et al.Capecitabine monotherapy for recurrent and metastatic nasopharyngeal cancer[J].Jpn J Clin Oncol,2008,38(4):244–249.

[8]Phase II study of capecitabine as palliative treatment for patients with recurrent and metastatic squamous head and neck cancer after previous platinum-based treatment[J].British Journal of Cacner,2010,102,1687-1691.

[9]Sheen TS,Huang YT,Chang YL,et al.EpsteinBarr virusencoded latent membrane protein 1 co expressed with epidermal growth factor receptor in nasopharyngeal carcinoma[J].Jpn J Cancer Res,1999,90(2):1285-1290.

[10]Gaffney DK,Haslam D,Tsodikov A,et al.Epidermal growth factor receptor (EGFR)and vascular endothelial growth factor(VEGF)negatively affect overall survival in carcinoma of the cervix treated with radiotherapy[J].Int J Radia Oncol Biol Phys,2003,56(4):922-928.

[11] Expert consensus on comprehensive treatment of head and neck tumors. Chinese Journal of Otorhinolaryngology Head and Neck Surgery, 2010, 45(7): 535-541.

[12]Chan, Hsu, Goh, et al.Multicenter, phase II study of cetuximab in combination with carboplatin in patients with recurrent or metastatic nasopharyngeal carcinoma [J].J Clin Oncol, 2005,23:3568-3576.

[13]Xu T,et al.Cetuximab in combination with chemoradiotherapy in the treatment of recurrent and/or metastatic nasopharyngeal carcinoma.Anti-Cancer Drugs, 2016, 27:66-70.

[14]Chen BJ, Chapuy B, Ouyang J, et al.PD-L1 expression is characteristic of a subset of aggressive B-cell lymphomas and virus-associated malignancies[J].Clin Cancer Rearch, 2013,19(13):3462-3473.

[15]Fang W,Zhang J,Hong S,et al.EBV-driven LMP1 and IFN-γup-regulate PD-L1 in nasopharyngeal carcinoma:Implications for oncotargeted therapy [J]. Oncotarget, 2014,5(23):12189-12202.

[16]Zhang J,Fang W,Qin T,et al.Co-expression of PD-1 and PD-L1 predicts poor outcome in nasopharyngeal carcinoma[J].Medical Oncology,2015,32(3):86.

[17]Chiun Hsu,Se-Hoon Lee,Samuel Ejad,,et al.Safety and antitumor activity of Pembrolizumab in patients with programmed death-ligand 1–positive nasopharyngeal carcinoma:results of the KEYNOTE-028 study[J].JCO.2017,73:3675.

[18]Krishna SM,James S,Balaram P.Expression of VEGF as prognosticator in primary nasopharyngeal cancer and its relation to EBV Statue[J].Virus Res,2006, 115(1):85-90.

[19]Lee NY,et al.Addition of bevacizumab to standard chemoradiation for locoregionally advanced nasopharyngeal carcinoma:a phase 2 multi-institutional trial.Lancet Oncol,2012,13:172-80.

[20]Elser C,et al.Phase II Trial of Sorafenib in Patients With Recurrent or Metastatic Squamous Cell Carcinoma of the Head and Neck or Nasopharyngeal Carcinoma.J Clin Oncol,2007,25:3766-3773.

[21]Xue C,et al.Phase II study of sorafenib in combination with cisplatin and 5-fluorouracil to treat recurrent or metastatic nasopharyngeal carcinoma.Annals of Oncology,2013,24:1055-1061.

[22]Hui EP,et al.Hemorrhagic complications in a phase II study of sunitinib in patients of nasopharyngeal carcinoma who has previously received high-dose radiation.Annals of Oncology,2011,22:1280-1287.

[23] Li B et al. A phase-II clinical study on Endostar-chemotherapy combined treatment for metastatic nasopharyngeal carcinoma. *Chinese Journal of Clinical Oncology*, 2012, 39(24): 2022-2025.

[24]Shukui Qin,et al.Initial dose of apatinib in Chinese patients with chemotherapy-refractory advanced or metastatic adenocarcinoma of stomach or gastroesophageal junction in third-or later-line setting:500mg or 850mg?2018 Gastrointe stinal Cancer Symposium.

[25]QX Peng,et al.Apatinib inhibits VEGFR-2 and angiogenesis in an in vivo murine model of nasopharyngeal carcinoma.Oncotarget , 2017,8 (32) :52813-52822.

[26]YS Lin,et al.Overwhelming rapid metabolic and structural response to apatinib in radioiodine refractory differentiated thyroid cancer.Oncotarget, 2017,8(26):42252.

[27]XH He,et al.A pilot study of Apatinib in heavily pretreated metastatic adenocarcinoma of the head and neck.2017 ESMO.

[28]Guopei Zhu,et al.Phase II study of apatinib,a novel VEGFR inhibitor in patients with recurrent and/or metastatic adenoid carcinoma of the head and neck:Preliminary results.2018 AHNS-ASTRO-ASCO.

#

# Appendix 1 AJCC/UICC 7th Edition TNM Staging

Nasopharynx (T)

T1 Tumor only in the nasopharynx or invades the oropharynx and/or nasal cavity, and does not invade the parapharyngeal space

T2 Tumor invades the parapharyngeal space

T3 Tumor invades the skull base and/or paranasal sinus

T4 Tumor invades the intracalvarium, cranial nerves, laryngopharynx, eye socket or infratemporal fossa/masticatory muscle space

Regional lymph nodes (N)

N1 Metastasis of unilateral or bilateral retropharyngeal lymph nodes, with the maximum diameter ≤ 6 cm

Metastasis of unilateral cervical lymph nodes, of the maximum diameter ≤ 6 cm and above the supraclavicular fossa

N2 Metastasis of bilateral cervical lymph node, with the maximum diameter ≤ 6 cm and above the supraclavicular fossa

N3a The maximum diameter > 6 cm

N3b Metastasis of supraclavicular lymph nodes

Distant metastasis (M)

M0 No distant metastasis

M1 With Distant metastasis

Overall Staging

Stage 0 Tis N0 M0

Stage I T1 N0 M0

Stage II T1 N1 M0

T2 N0 M0

T2 N1 M0

Stage III T3 N0, N1 M0

T1, T2, T3 N2 M0

Stage IVA T4 N0, N1, N2 M0

Stage IVB Any T N3 M0

Stage IVC Any T Any N M1

# Appendix 2 ECOG PS Scoring

| **Score** | **ECOG performance status** |
| --- | --- |
| 0 | Fully active, able to carry on all pre-disease performance without restriction |
| 1 | Restricted in physically strenuous activity but ambulatory and able to carry out work of a light or sedentary nature, for example, light house work, office work |
| 2 | Ambulatory and capable of all self-care but unable to carry out any work activities; Up and about more than 50% of waking hours |
| 3 | Capable of only limited self-care, confined to bed or chair more than 50% of waking hours |
| 4 | Completely disabled; Cannot carry on any self-care; Totally confined to bed or chair |

# Appendix 3 Flow Chart of the Study

| **Item** | **Within 4 weeks before treatment** | **Within 1 week before treatment** | **Treatment cycle** | | | | | | | | | | |
| --- | --- | --- | --- | --- | --- | --- | --- | --- | --- | --- | --- | --- | --- |
|  |  |  | **Cycle 1** | | | **Cycle 2** | **Cycle 3** | **Cycle 4** | **Cycle 5** | **Cycle 6** | **Odd cycles** | **Even cycles** | **End of treatment/withdrawal** |
|  |  |  | 1 w | 2 w | 3 w |  |  |  |  |  |  |  |  |
| Sign the Informed Consent form | **ⅹ** |  |  |  |  |  |  |  |  |  |  |  |  |
| Treatment history | **ⅹ** |  |  |  |  |  |  |  |  |  |  |  |  |
| Vital signs | **ⅹ** | **ⅹ** |  |  | **ⅹ** | **ⅹ** | * | # | * | # | * | # | **ⅹ** |
| ECOG PS score | **ⅹ** | **ⅹ** |  |  | **ⅹ** | **ⅹ** | * | # | * | # | * | # | **ⅹ** |
| Blood pressure a | **ⅹ** | **ⅹ** | **3** | **3** | **3** | * | * | * | * | * | * | * | **ⅹ** |
| Blood sample b |  | **ⅹ** |  |  | **ⅹ** | **ⅹ** | * | # | * | # | * | # | **ⅹ** |
| Routine blood tests |  | **ⅹ** |  |  | **ⅹ** | **ⅹ** | * | # | * | # | * | # | **ⅹ** |
| Hepatic and renal function, electrolyte |  | **ⅹ** |  |  | **ⅹ** | **ⅹ** | * | # | * | # | * | # | **ⅹ** |
| EB virus (by PCR) |  | **ⅹ** |  |  | **ⅹ** | **ⅹ** | * | # | * | # | * | # | **ⅹ** |
| Nasopharynx + neck CT/MRI c | **ⅹ** |  |  |  |  | **ⅹ** |  | # |  | # |  | # | **ⅹ** |
| Distant metastasis examination d | **ⅹ** |  |  |  |  | **ⅹ** |  | # |  | # |  | # | **ⅹ** |
| Urine routines e |  | **ⅹ** |  |  | **ⅹ** | **ⅹ** | * | # | * | # | * | # | **ⅹ** |
| Fecal routines (OB) |  | **ⅹ** |  |  | **ⅹ** | **ⅹ** | * | # | * | # | * | # | **ⅹ** |
| Coagulation function |  | **ⅹ** |  |  | **ⅹ** | **ⅹ** | * | # | * | # | * | # | **ⅹ** |
| ECG f |  | **ⅹ** |  |  | **ⅹ** | **ⅹ** | * | # | * | # | * | # | **ⅹ** |
| UCG | **ⅹ** |  |  |  |  |  |  |  |  |  |  |  | **ⅹ** |
| Pregnancy test |  | **ⅹ** |  |  |  |  |  |  |  |  |  |  | **ⅹ** |
| Quality of life score |  | **ⅹ** |  |  | **ⅹ** | **ⅹ** | * | # | * | # | * | # | **ⅹ** |
| Adverse events | **ⅹ** | **ⅹ** | **ⅹ** | **ⅹ** | **ⅹ** | **ⅹ** | * | # | * | # | * | # | **ⅹ** |
| Concomitant medication | **ⅹ** | **ⅹ** | **ⅹ** | **ⅹ** | **ⅹ** | **ⅹ** | * | # | * | # | * | # | **ⅹ** |
| Combination Medication | **ⅹ** | **ⅹ** | **ⅹ** | **ⅹ** | **ⅹ** | **ⅹ** | * | # | * | # | * | # | **ⅹ** |

Note: **1) All observation indicators and examination time should not be affected by the duration of drug suspension, and all relevant examinations (including imaging examinations) during each course of treatment should be performed following the flow chart; 2) It is recommended to suspend the drug for 2 to 3 days before puncture or forceps biopsy (if necessary for the patient) as anti-vascular drugs may affect wound healing (because the drug has a half-life period of 9 hours, and 97% of it can be removed from the body after 5 half-life periods), and restore the drug after the wound is healed.**

*: Same as Cycle 1; #: Same as Cycle 2; a: 3 means to measure the blood pressure once every three days; b: Serum VEGF-A/C and VEGFR-2 levels; c: Re-check every two cycles, and follow up every three months after PD/withdrawal; d: Check for hepatic, pulmonary or osseous metastasis by routine chest imaging, liver B-mode ultrasound and bone scanning examinations. If the physician-in-charge considers it appropriate, CT/MRI/PET-CT can be used instead; e: If the urine routines show urine protein of 2+, please perform the 24-hour urine protein quantitative examination and adjust the dose of the drug according to the examination result; f: Supplementary UCG can be performed if there are abnormal ECG findings and the patient has suspected clinical symptoms, or the physician-in-charge considers it necessary.
